# Maternal immune activation as an epidemiological risk factor for neurodevelopmental disorders: Considerations of timing, severity, individual differences, and sex in human and rodent studies

**DOI:** 10.3389/fnins.2023.1135559

**Published:** 2023-04-13

**Authors:** Mary Beth Hall, Daria E. Willis, Elina L. Rodriguez, Jaclyn M. Schwarz

**Affiliations:** Schwarz Lab, Department of Psychological and Brain Sciences, University of Delaware, Newark, DE, United States

**Keywords:** neurodevelopmental disorders, maternal immune activation, perinatal period, development, individual differences, sex differences, autism spectrum disorder, schizophrenia

## Abstract

Epidemiological evidence suggests that one’s risk of being diagnosed with a neurodevelopmental disorder (NDD)—such as autism, ADHD, or schizophrenia—increases significantly if their mother had a viral or bacterial infection during the first or second trimester of pregnancy. Despite this well-known data, little is known about how developing neural systems are perturbed by events such as early-life immune activation. One theory is that the maternal immune response disrupts neural processes important for typical fetal and postnatal development, which can subsequently result in specific and overlapping behavioral phenotypes in offspring, characteristic of NDDs. As such, rodent models of maternal immune activation (MIA) have been useful in elucidating neural mechanisms that may become dysregulated by MIA. This review will start with an up-to-date and in-depth, critical summary of epidemiological data in humans, examining the association between different types of MIA and NDD outcomes in offspring. Thereafter, we will summarize common rodent models of MIA and discuss their relevance to the human epidemiological data. Finally, we will highlight other factors that may interact with or impact MIA and its associated risk for NDDs, and emphasize the importance for researchers to consider these when designing future human and rodent studies. These points to consider include: the sex of the offspring, the developmental timing of the immune challenge, and other factors that may contribute to individual variability in neural and behavioral responses to MIA, such as genetics, parental age, the gut microbiome, prenatal stress, and placental buffering.

## 1. Introduction

According to the Centers for Disease Control and Prevention, the prevalence of neurodevelopmental disorders (NDDs) in the United States is 13.87% and yet the etiology of these disorders is not well understood. This rate has increased by about 9.5% in the last decade (Zablotsky et al., 2019), likely because our understanding of and ability to effectively diagnose various NDDs has improved over time. NDDs are similarly prevalent across most countries throughout the world, although the rates may vary due to socioeconomic factors, awareness, and diagnostic methods within each country (Chiarotti and Venerosi, 2020). Common epidemiological trends associated with NDDs include: the general age of onset within each disorder, symptom manifestation within each disorder, sex bias in the prevalence of certain NDDs, as well as the possible risk factors associated with many NDDs.

Epidemiological data suggest that genetic risk provides a foundation upon which other factors may precipitate or enhance the risk for many NDDs (Zawadzka et al., 2021). One of those other risk factors is prenatal infection associated with maternal immune activation, which slightly but significantly increases the risk of various NDDs. Maternal immune activation (MIA) is a term used in epidemiological studies that typically refers to maternal exposure to, or infection with, various immunogens (i.e., viral, bacterial, parasitic) during pregnancy. Some human studies have also considered increased levels of immune-related molecules (i.e., cytokines, chemokines) to serve as indicators of MIA. Animal studies are also commonly used to model MIA either *via* direct infection (i.e., of a virus, bacteria, or parasite) or *via* stimulation of the immune system (in the absence of infection) by utilizing a viral or bacterial mimetic, immune-related molecules, or other environmental stressors that are known to activate the immune system. Rodent models of MIA have been used extensively to model and better understand how activation of the immune system during gestation may affect the development of neurobiological systems underlying NDDs. Neuroscientists have only just begun to understand how the maturation of certain structures in the brain allows for the emergence of particular behaviors at specific ages (see Albani et al., 2014 for review). As such, it is still not well-understood how developing neural circuits or systems are disrupted by events such as immune activation that, in turn, increase the risk of NDDs or explain the underlying etiology of their symptoms.

In this review, we will (1) summarize epidemiological evidence that supports the role of MIA in the risk for NDDs, with a critical eye towards new emerging trends in the data, (2) introduce commonly used rodent models of MIA and their relevance for studying human NDDs, and (3) assess additional factors that should be considered when studying NDDs in rodents, including timing and severity of infection, sex differences in vulnerability for and symptomatology of NDDs, and individual differences associated with the maternal immune response. Thus, the overall goal of this review paper is to evaluate the epidemiological link between MIA and NDDs in order to identify factors that should be considered when designing future human and rodent studies. By considering additional dimensional criteria in their experimental design, researchers may begin to better address the immunological and neurobiological causes of NDDs and effectively identify possible treatments or therapies.

## 2. What are neurodevelopmental disorders?

The term “neurodevelopmental disorders” (NDDs) was first introduced as a diagnostic category in the *DSM-5*, to replace the more general term “developmental disorders” that was introduced in the *DSM-III* (Morris-Rosendahl and Crocq, 2020). These disorders affect one or several areas of development, including language, motor, social, and learning skills. More specifically, NDDs are a group of conditions that produce impairments of functioning during development and are associated with a *known* early-life medical, environmental, or genetic risk factor (Morris-Rosendahl and Crocq, 2020). Examples of NDDs defined in the *DSM-5* include, but are not limited to, autism spectrum disorder (ASD), attention-deficit/hyperactivity disorder (ADHD), intellectual disability (ID), and communication disorders. In 2019 and 2020 in the United States, the prevalence of ADHD was ~8.5%, ASD was ~2.9%, intellectual disability (ID) was ~1.4%, and various learning disabilities (LD) was ~6.4% (Yang et al., 2022). Notably, a higher prevalence of ADHD, ASD, ID, and LD have all been observed in boys relative to girls aged 3–17 (Yang et al., 2022). Schizophrenia is also considered by many researchers and clinicians to fall under the category of NDDs even though it is not defined as such in the *DSM-5*. This is because while the positive symptoms of schizophrenia (hallucinations, disorganized speech, etc.) typically manifest first during late adolescence, the etiology of these symptoms likely result from events that occur during perinatal or early postnatal development (Brašić and Holland, 2007; Fatemi and Folsom, 2009; Rapoport et al., 2012; Heyer and Meredith, 2017).

Interestingly, there are often overlapping symptoms experienced by people diagnosed with various NDDs. These include cognitive and learning disabilities (Pope and Kern, 2006; Gold et al., 2008; Cicero et al., 2014; Wang et al., 2017; Banker et al., 2021), decreased social behaviors (Nijmeijer et al., 2008; Hooley, 2010; Uekermann et al., 2010; Savla et al., 2013; Staikova et al., 2013; Supekar et al., 2013; Perepa, 2014; Cotter et al., 2018; Porcelli et al., 2019), altered sleep patterns or disrupted circadian rhythms (Williams et al., 2004; Cohrs, 2008; Konofal et al., 2010; Stein et al., 2012; Hvolby, 2014; Myles et al., 2016; Kaskie et al., 2017; Shelton and Malow, 2021), as well as metabolic or gastrointestinal disturbances (Richardson and Ross, 2000; Singh et al., 2020; Oyarzábal et al., 2021). It is important to note that symptoms shared across different NDDs can manifest differently based on the specific disorder and the individual person. For example, people diagnosed with ADHD, ASD, or schizophrenia may experience dysregulated sleep or circadian rhythm cycles, which can range from having delayed onset of sleep and melatonin peak to having decreased efficiency and total amount of sleep (Williams et al., 2004; Cohrs, 2008; Konofal et al., 2010; Stein et al., 2012; Shelton and Malow, 2021). People diagnosed with ADHD or schizophrenia may also experience sleep apnea or obstructed breathing during sleep (Konofal et al., 2010; Hvolby, 2014; Myles et al., 2016; Kaskie et al., 2017). Furthermore, particular modes of learning are differentially affected across NDDs. Schizophrenia is associated with difficulties in verbal learning, learning that requires self-correction, learning that happens on a rapid timescale, and reward or reinforcement learning (Pope and Kern, 2006; Gold et al., 2008; Cicero et al., 2014), whereas ASD is associated with impairments in spatial working memory, spatial navigation and reasoning, and memory retrieval tasks (Wang et al., 2017; Banker et al., 2021). Thus, the phenotype of a “shared” NDD symptom may manifest differently depending on the distinct NDD of that individual.

It is currently unknown whether the neurobiology contributing to the overarching “shared” NDD symptoms is similarly impacted in humans across different disorders (see De Lacy and King, 2013 for review of neurobiological studies underlying ASD and schizophrenia). On one hand, it is possible that across different NDDs, a shared symptom may be caused by a disturbance in a *shared* neurobiological process. One hypothesis is that dysfunction across NDDs may be driven by alterations in the excitatory/inhibitory balance in the brain (Pearce, 2001; Foss-Feig et al., 2017). Additionally, Nair et al. (2020) theorize that reduced social behaviors in adolescents with ASD or psychosis are linked to disruptions in the default mode network. On the other hand, it is possible that for different NDDs, a shared symptom could be caused by a disturbance in *distinct* neurobiological processes. For instance, evidence suggests that different patterns of underconnectivity of long-range axons between multiple brain regions and of overconnectivity of short-range axons within a brain region are implicated in the pathogenesis of ASD, ADHD, and schizophrenia (see De Lacy and King, 2013 and Kern et al., 2015 for information about the underlying mechanisms and implications of these altered connectivity patterns). Specifically, underconnectivity between frontal cortex and posterior brain areas is associated with ASD whereas underconnectivity between parietal cortex and the cerebellum is associated with ADHD (Kern et al., 2015). In this case, even though a similar mechanism of dysfunction may be similar across disorders, the specific characteristics (and likely the etiology) of the disruption are distinct for each disorder. Finally, dysregulation of the immune system following MIA may contribute to the etiology of many NDDs, in that it elicits a core set of symptoms that are similar to sickness behavior and are consistent across different NDDs, including cognitive or learning deficits, decreased social behavior, metabolic or gastrointestinal disturbances, and dysregulated sleep.

In summary, age of onset, cause, severity, etiology, and manifestation of symptoms can be different amongst individuals, even within one type of NDD (e.g., ASD). This variability is not specific to any one NDD or to the class of NDDs in general, rather it applies to many mental health and psychiatric disorders where the diagnostic criteria reflect a collection of symptoms that often overlap. This variability and overlap in symptomatology across various disorders led the National Institutes of Mental Health (NIMH) to create the Research Domain Criteria (RDoC) framework, which focuses on a dimensional rather than categorical approach to preclinical research. The RDoC encourages researchers to study specific criteria (i.e., dimensions) related to a disorder (e.g., risk factors such as MIA or symptoms such as specific types of learning deficits), rather than attempting to model the entirety of a disorder. By focusing experimental designs -- of both epidemiological studies and rodent models -- on studying specific criteria related to an NDD, we may gain a better understanding of the underlying circuits and mechanisms pertaining to multiple NDDs that exhibit that criterion as a symptom or risk factor. This framework also highlights the need for researchers to consider *individual differences* in the expression of specific symptoms (or RDoC dimensions) when investigating risk factors for NDDs, which may ultimately provide us with a better understanding of how the symptoms, ontogeny, and severity of NDDs can be so distinct between one case to the next. There are many types of environmental factors and stressors that commonly and strongly predict the risk of NDDs, including genetic factors (Carter, 2009; McCarroll and Hyman, 2013), sex (Bargiela et al., 2016; Lai, et al., 2017), parental age, stress, diet, as well as prenatal and birth complications (summarized in Carlsson et al., 2021). That said, we will discuss the epidemiological evidence supporting that MIA is a well-known risk factor for many NDDs.

## 3. Maternal immune activation is an epidemiological risk factor for neurodevelopmental disorders

The developing brain is uniquely vulnerable to environmental insults and infections that can adversely impact the neurodevelopmental trajectory and ontogeny of behavior later in life (Bale, 2009; Deverman and Patterson, 2009; Schwarz and Bilbo, 2011b). Interestingly, the immune system has an important role in the various processes of typical neural development (Schwarz and Bilbo, 2011b; Tanabe and Yamashita, 2018; Zengeler and Lukens, 2021). Epidemiological data support that MIA increases the risk for NDDs in offspring. It is important to note that in both human and animal studies, MIA typically refers to any immune challenge that occurs during pregnancy. However, animal studies modeling gestational development can also encompass the perinatal period more broadly—occuring during gestation or around the time of birth—because the first 2 weeks of neonatal development in rodent pups is roughly equivalent to the third trimester of fetal development in humans (Guma et al., 2019). More specifically, third trimester neurodevelopmental processes such as immunogenesis, apoptosis, and synaptogenesis, occur during gestation in humans but continue post-birth in rodents (Estes and McAllister, 2016).

In humans, cohort and case–control studies are common experimental designs used to examine the relationship between MIA and NDD diagnosis (Song and Chung, 2010). Cohort studies first identify people that were exposed to an infectious agent during a specific time, and then either prospectively or retrospectively examine the likelihood that they are diagnosed with the disorder being studied. On the other hand, case–control studies first identify people diagnosed with the disorder of interest, and then retrospectively determine if they experienced an associated exposure or risk factor. Incidences of infection are typically confirmed *via* self-report, old medical records, or serological confirmation of infection. One limitation of these human studies is the necessity of an observational design, which prevents us from fully understanding the causal relationship between MIA and symptoms of NDDs. This highlights the importance of basic biomedical research and animal models to decipher the specific link between MIA and the ontogeny of NDDs, with the additional goal of identifying the underlying molecular, cellular, or neural circuit mechanisms or disruptions. Nevertheless, epidemiological studies of maternal exposure to various pathogens and environmental triggers—particularly viral and bacterial infections—during gestation provide some of the strongest data linking MIA and the risk of NDDs (see Han et al., 2021 for a more comprehensive review).

### 3.1. The epidemiological evidence with a focus on infection type, severity, febrile response, and medications

General infections during pregnancy have been associated with increased risk for ASD and schizophrenia in offspring (Nielsen et al., 2013; Jiang et al., 2016; Zhou et al., 2021). More specifically, viral and bacterial infections during gestation are well-associated with later NDD diagnosis. Various viral infections during pregnancy are linked with ASD diagnosis (e.g., rubella, congenital cytomegalovirus, influenza) and schizophrenia diagnosis (e.g., influenza, rubella, Herpes simplex virus type 2 diagnosis) in offspring (see review articles: Boksa, 2008; Brown, 2012; Ornoy et al., 2015; Shuid et al., 2021; Cheslack-Postava and Brown, 2022; Massarali et al., 2022). Certain bacterial infections during gestation are also associated with ASD (e.g., urinary tract infection, genital infections) and schizophrenia (e.g., respiratory infections, pyelonephritis, and genital/reproductive infections) diagnoses in offspring (see review articles: Boksa, 2008; Brown, 2012; Cheslack-Postava and Brown, 2022; Massarali et al., 2022). Parasitic infections during pregnancy, particularly *Toxoplasmosis gondii* (*T. gondii*), have also been linked to schizophrenia in offspring (Khandaker et al., 2013; Cheslack-Postava and Brown, 2022). Some case–control studies have shown that individuals with schizophrenia were more likely to have IgG antibodies against *T. gondii* (Hamidinejat et al., 2010), be exposed to maternal Toxoplasma IgG antibodies during gestation (titer ≥1: 128) (Brown et al., 2005), or have increased levels of IgG antibodies against *T. gondii* as infants (Mortensen et al., 2007). The next subsections will discuss specific types of infections and factors most notably associated with the risk of NDDs, with an emphasis on ASD and schizophrenia.

#### 3.1.1. Rubella

During the 1960s there was a rubella epidemic in the United States that resulted in various pregnancy and birth complications as well as physical and cognitive birth defects in the affected infants (Lindquist et al., 1965; Chess et al., 1979; Berger et al., 2011). While many cases of rubella infection have since been prevented by vaccination, rubella is estimated to still affect around 5% of pregnant persons worldwide (Berger et al., 2011; Hutton, 2016). Early links between rubella and ASD were identified from a New York cohort study of children in the United States that were part of the Rubella Birth Defect Evaluation Project (RBDEP). This study identified a significant correlation between congenital rubella syndrome (CRS) and autism diagnosis during childhood (Chess et al., 1979). Of particular note, CRS and ASD seem to overlap in their manifestation and symptomatology (Desmond et al., 1969; Swisher and Swisher, 1975; Hutton, 2016; Mawson and Croft, 2019). One mechanism thought to underlie the link between maternal rubella infection, particularly during the first trimester, and ASD risk in offspring is *via* liver dysfunction resulting in fetal exposure to high levels of vitamin A, which can be toxic to brain and other tissues of the developing fetus (Mawson and Croft, 2019).

Another cohort study of the RBDEP found that prenatal rubella exposure was associated with risk for nonaffective psychosis in young adulthood, regardless of hearing loss (Brown et al., 2000a). This association held true during a follow-up study with an updated assessment that allowed for a diagnosis of schizophrenia (Brown et al., 2001), which provided evidence that prenatal rubella is linked with an increased risk for schizophrenia in young adulthood. CRS and schizophrenia also overlap in brain dysmorphology, with both groups having reduced cortical gray matter volume and enlarged lateral ventricle volume, when adjusted for age and head size (Lim et al., 1995). In all, additional research still needs to be conducted to determine the underlying characteristics of prenatal rubella infection that may contribute to symptoms of ASD and schizophrenia, teased apart from other symptoms more specific to CRS.

#### 3.1.2. Bacterial infections

In a Swedish cohort study, bacterial infection, not associated with a particular trimester of pregnancy, was linked with ASD without comorbid intellectual disability, ID (Lee et al., 2015). A significant association between ASD diagnosis and general bacterial infection during the third trimester was also reported in a Taiwanese case–control study (Fang et al., 2015). A meta-analysis similarly found that bacterial infection, particularly during the second or third trimester, was associated with ASD in offspring (Jiang et al., 2016). Moreover, bacterial infections requiring hospitalization during the second trimester (most commonly including urinary tract infection and genital infection) were linked to ASD diagnosis in a Danish cohort study (Atladóttir et al., 2010). Similarly, in a California case–control study in the United States, bacterial infections (such as urinary tract infection, amniotic infection at delivery, and major puerperal infection) diagnosed during a hospital stay, particularly during the third trimester of pregnancy, were significantly associated with risk for ASD in offspring (Zerbo et al., 2015). Additionally, in a Danish cohort study, genitourinary infections during weeks 33–36 of the third trimester were significantly linked with increased risk for ADHD in offspring (Werenberg Dreier et al., 2016).

Furthermore, a meta-analysis found increased risk of psychosis in offspring linked to general bacterial infections during pregnancy (Zhou et al., 2021). In a Danish cohort study, exposure to bacterial infection (including sinusitis, tonsillitis, pneumonia, cystitis, pyelonephritis, and bacterial venereal infection) during the first trimester of pregnancy was associated with an elevated risk for schizophrenia in offspring (Sørensen et al., 2009). Exposure to maternal genital/reproductive (G/R) infections during the periconceptual period (such as endometritis, cervicitis, pelvic inflammatory disease, vaginitis, syphilis, condylomata, “venereal disease,” and gonorrhea) was also linked with increased risk for schizophrenia in offspring (Babulas et al., 2006). Further, pyelonephritis infection (kidney infection) that required hospitalization was associated with schizophrenia in offspring, but notably, only when there was a family history of psychosis (Clarke et al., 2009). Similarly, in a Swedish population-based cohort study, maternal infection during pregnancy was associated with later psychosis in offspring, only when there was also parental history of a psychiatric disorder (Blomström et al., 2016). While much of the above evidence supports that maternal bacterial infection increases the risk of NDDs, these last two studies, in particular, suggest that there may alternatively be an underlying susceptibility to perinatal infection in families with a history of NDDs, a concept that we will discuss in greater detail below.

#### 3.1.3. Influenza

Influenza during pregnancy has been linked to an increased risk of schizophrenia in affected offspring (Khandaker et al., 2013). Two cohort studies reported an increased risk of schizophrenia associated with serologically confirmed maternal influenza infection, although this increased risk was ultimately not statistically significant (Brown et al., 2004; Ellman et al., 2009). In a prediction model, the number of influenza deaths in the general population of England was significantly associated with risk of schizophrenia in offspring that were in their 6th or 7th month of gestation at the time (Sham et al., 1992). Similarly, the number of influenza infections in the general population of Denmark were linked with schizophrenia risk in offspring that were in their 6th month of gestation at that time (Barr et al., 1990; Takei et al., 1996). Moreover, using data from influenza epidemics in France between 1949 and 1981, Limosin et al. (2003) also reported that adults with schizophrenia were more likely to have been exposed to influenza during the 5th month of gestation as compared to controls.

Often, a major limitation of these studies is the lack of direct link between prenatal influenza exposure and schizophrenia outcomes within the same subjects, although a few studies *have* established this link. In a study examining outcomes of the 1957 influenza epidemic in Finland, admissions into psychiatric hospitals for schizophrenia in offspring was associated with a second-trimester gestational age at the time of the epidemic (Mednick et al., 1988). Prenatal infections were later confirmed *via* medical records, supporting that influenza exposure during the second trimester was associated with an increased risk for schizophrenia as compared to infection during the first or third trimesters (Mednick et al., 1994). Further, a Californian cohort study in the United States found that respiratory infections during the second trimester of pregnancy were significantly associated with schizophrenia spectrum disorder diagnosis in offspring (Brown et al., 2000b).

There is perhaps more limited evidence supporting an association between influenza infection during gestation, not specifically linked to a specific trimester, and increased risk for ASD (Atladóttir et al., 2012). In a United States study using a large patient dataset within Kaiser Permanente healthcare network in Northern California (Zerbo et al., 2017), and in a Norwegian cohort study (Mahic et al., 2017), there were no significant associations found between influenza infection during pregnancy and ASD diagnosis. On the other hand, influenza infection during the second trimester was significantly associated with risk for ASD in a Boston cohort study in the United States (Holingue et al., 2020). Interestingly, this association was only true when antibiotics were *not* taken at any point during pregnancy, and not necessarily that they were only avoided at the specific time of infection (Holingue et al., 2020). In all, there is limited and conflicting evidence for the link between prenatal influenza and NDDs, particularly schizophrenia and ASD, which suggests that the association may be more complex than initially reported.

#### 3.1.4. Fever during pregnancy

Even though ASD and developmental delays were not associated with general influenza exposure in the Northern California cohort, the incidence of ASD and NDDs in offspring *were* significantly associated with fever during pregnancy in an earlier study (Zerbo et al., 2013). As expected, this fever-associated risk for ASD was attenuated in people that reported taking antipyretics to reduce their fever during pregnancy (Zerbo et al., 2013). Supporting these data, a meta-analysis examining the relationship between prenatal immune activation and ASD diagnosis found a significant association between maternal fever and ASD diagnosis in offspring; this association was not significant for prenatal infections without fever (Tioleco et al., 2021). A United States case–control study also found that there was a significant association between ASD risk and having a fever during the second trimester, even though there was no association between general prenatal infection and ASD risk (Croen et al., 2019). Thus, it is possible that links between maternal influenza during pregnancy and ASD risk may be obscured by unmeasured febrile response and unreported medication use (either antibiotics or antipyretics) in retrospective epidemiological studies, or through the lack of inclusion of such criteria in the original study design or analysis.

Furthermore, there is some evidence that maternal febrile response to influenza infection during pregnancy may be an important factor associated with increased risk for schizophrenia in offspring (Edwards, 2007). A Finnish cohort study found an increased odds ratio for schizophrenia in offspring that was associated with maternal fever during pregnancy, however the data were not statistically significant (Jones et al., 1998). Moreover, a Danish cohort study found that exposure to infections or maternal fever during gestation was associated with offspring having one psychosis-like experience by 11-years of age (Dreier et al., 2018). On another note, a Danish cohort study identified an association between maternal fever during weeks 9–12 of the first trimester and ADHD risk in offspring (Werenberg Dreier et al., 2016). Overall, it seems that the febrile response to infection during pregnancy or during parturition is linked with risk for NDDs in offspring and may serve as a potential mechanism to disrupt neurobiological development in the fetus and/or child.

#### 3.1.5. Severity of infection

An additional nuance to the data presented thus far is the severity and duration of an infection or febrile response during pregnancy. A Danish cohort study reported that febrile episodes lasting more than 1 week, and that occurred prior to 32 weeks of gestation (roughly mid-third trimester), were associated with a threefold increase in risk for ASD in offspring (Atladóttir et al., 2012). Supporting this evidence, a study using data from the Norwegian Mother and Child Cohort Study (Magnus, et al., 2006) and Autism Birth Cohort Study (Stoltenberg, et al., 2010) found that maternal fever during the second trimester was associated with increased risk for ASD, and this risk was augmented with increased number of febrile episodes (Hornig et al., 2018).

Furthermore, a Swedish cohort study found an increased risk of ASD diagnosis associated with exposure to severe types of perinatal infections (such as sepsis, pneumonia, pyelonephritis, meningitis or encephalitis, influenza, and chorioamnionitis) versus a non-severe urinary tract infection (Al-Haddad et al., 2019). Having two or more infections during pregnancy, particularly during the third trimester, was also associated with increased risk for ASD (Zerbo et al., 2015). Along these lines, a Danish cohort study found that hospital admissions for viral infection during the first trimester (including influenza, gastroenteritis, rubella, etc.) and for bacterial infection (including urinary tract and genital infections) during the second trimester were significantly associated with an increased risk for ASD diagnosis (Atladóttir et al., 2010). Similarly, a meta-analysis showed that infection during pregnancy that required hospitalization was associated with increased risk for ASD, particularly during the first or second trimester of pregnancy (Jiang et al., 2016).

Psychosis risk in offspring has also been linked to hospital treatment for maternal infection during pregnancy (Zhou et al., 2021). Interestingly, schizophrenia in offspring has also been associated with both maternal and *paternal infections* that specifically required a visit to the hospital, regardless of whether the visit occurred before, during, or after pregnancy (Nielsen et al., 2013). This suggests that familial history of prior illness or general infection may also be an important factor to consider in the risk for NDDs, which we will discuss more later in this review. Overall, these data indicate that, during gestation, the magnitude of the febrile response and the severity of infection may be specific factors important for consideration in the risk of ASD and schizophrenia diagnoses.

#### 3.1.6. Cytokine and chemokine expression during pregnancy

As originally proposed by Dr. Paul Patterson and confirmed by many colleagues since, one mechanism by which influenza (or other infections) increases the risk of NDDs in offspring may be *via* the maternal immune response and its associated circulating cytokines, rather than *via* a direct infection of the fetus itself (Shi et al., 2005; Mahic et al., 2017). In the past few decades, human epidemiological studies have provided further evidence of this idea. A Californian case–control study in the United States found that elevated levels of interferon (IFN)-γ, interleukin (IL)-4, and IL-5 in maternal serum mid-pregnancy were associated with increased ASD risk in offspring relative to the general population (Goines et al., 2011). Importantly, cytokine levels were adjusted for covariates during analysis, including gestational age at the time of specimen collection and maternal weight, age, ethnicity and country of birth. Similarly, a Danish case–control study found increased levels of tumor necrosis factor (TNF)-α and TNF-β in amniotic fluid (collected during screening or diagnostic amniocentesis procedures) of individuals later diagnosed with ASD (Abdallah et al., 2013). The researchers also found an association between ASD and elevated levels of IL-4, IL-10, and monocyte chemoattractant protein (MCP)-1 in the amniotic fluid of individuals born after 1993, which is when an updated diagnostic code for autism was introduced (Abdallah et al., 2012, 2013).

Moreover, a Philadelphia case–control study in the United States analyzed maternal serum samples that were collected during various prenatal visits or at birth and found that elevated levels of pro-inflammatory cytokines TNF-α, IL-1β, and IL-6 were associated with the diagnosis of a psychiatric disorder (i.e., schizophrenia, schizoaffective disorder, and major depression or bipolar disorder with psychosis) decades later in adult offspring (Allswede et al., 2020). This association was particularly true for samples collected during the first half of pregnancy but was not significant for those collected during the second half of pregnancy. Similarly, a Rhode Island study in the United States found increased levels of TNF-α and IL-8 in serum collected at parturition that were linked with reports of maternal infection during the third trimester (Buka et al., 2001). The elevation in TNF-α was specifically associated with increased risk for psychosis in offspring.

Overall, these studies support that activation of the immune system (*via* infection) induces a concert of cytokines and chemokines that circulate throughout the body to fight the invading pathogen. In doing so, these pleiotropic immune molecules have powerful effects on the body; they can pass through the placental barrier and enter the fetal compartment, or they can trigger cytokine production in the placenta itself, which can produce similarly powerful effects on the developing fetus (Hsiao and Patterson, 2012). This inflammatory response can thus drive alterations in the fetal brain by disrupting processes important for typical neural development, which may underlie later vulnerability to NDDs and their symptoms.

As mentioned previously, the immune system is highly involved in regulating numerous processes important for neural development. Microglia, the innate immune cells of the brain, are involved in the pruning and maturation of appropriate synapses during development *via* phagocytosis of axonal terminals and dendritic spines (Schafer et al., 2012). Exposure to early-life immune activation (e.g., MIA) compromises these functions, and can subsequently affect the number and function of microglia, disrupt synaptic maturation and pruning, and result in neural circuit remodeling and deficits in neural function and behavior (Paolicelli et al., 2011; Zhan et al., 2014; Tay et al., 2017). The consequences of this dysregulation may also extend beyond fetal development and affect neurodevelopmental processes during the postnatal period (Matcovitch-Natan et al., 2016; de Cossío et al., 2017), such as disrupting synapse/neural circuit formation important for the ontogeny of specific behavioral phenotypes (e.g., language acquisition, social behaviors, learning, etc.). Thus, rodent models of MIA and NDDs are especially important to help elucidate the neural and molecular processes that can become disrupted by immune activation during sensitive periods of development. In particular, many rodent models have focused on measuring or manipulating the specific cytokines produced during a maternal infection or immune challenge, in order to examine their effects on offspring brain and behavior throughout the lifespan. (For a more detailed review of neurobiological processes associated with NDDs that can become disrupted by MIA, see: Knuesel et al., 2014; Estes and McAllister, 2016; Bergdolt and Dunaevsky, 2019).

### 3.2. Considerations for future epidemiological studies

There is significant overall evidence suggesting that maternal infection with viruses or bacteria significantly increases the risk of various NDDs. ASD seems to be associated with exposure to viral infections during the first/second trimesters and bacterial infections or fever during the second/third trimesters of pregnancy. On the other hand, schizophrenia seems to be associated with viral infections during the second/third trimesters and bacterial infections during the first trimester of pregnancy. However, many epidemiological studies are unable to account for the trimester of infection in their findings, either due to limitations of the data collected or not having enough statistical power. Therefore, one cannot exclude the possibility that infections or febrile episodes during other phases of gestation are not also risk factors for these NDDs and their behavioral symptoms.

The characteristics, timing, and severity of the maternal immune response to infection seem to matter greatly and can vary depending on the type of infectious agent and whether a robust febrile response occurs. In turn, medications that attenuate febrile response or cytokine production (such as antipyretics or antibiotics) may lessen the risk of NDDs associated with prenatal infection and may also be strong determinants in the outcomes of fetal neurodevelopment that determine NDD risk. Intriguingly, prenatal acetaminophen use has been linked to increased risk for ADHD (Avella-Garcia et al., 2016; Liew et al., 2016), and another study linked to ASD diagnosis in children that also had hyperkinetic symptoms (Liew et al., 2014). Additional data is needed to examine how these specific aspects of an infection may be a driving factor for how the fetal immune system or neural development is altered or compromised. For instance, is a febrile response the driving factor? What is the contribution of the maternal peripheral immune response, or the placental immune response, or the fetal brain cytokine response to infection? In future human studies, serum or amniotic fluid samples should be collected and analyzed to better characterize the severity of the maternal or fetal immune and cytokine response, as originally proposed by Gilmore and Jarskog (1997). This may allow us to determine whether the immune response itself moderates the relationship between perinatal infection and the risk of NDDs in affected offspring. Moreover, the need for easily accessible serological and cell samples throughout gestation, in women with and without overt infection, will ultimately contribute to our better understanding of *how* the maternal immune reaction may be linked to NDDs.

Limited data from biological tissues and postmortem samples (Lintas et al., 2010) in humans has made it difficult to identify the biomarkers of disease that may be linked to MIA or perinatal infection. Human neuroimaging studies have begun to elucidate the association between elevated maternal cytokines during gestation and changes in brain structural and functional connectivity in offspring, which may or may not be associated with a particular NDD (see Guma et al., 2019 for data). The combined use of human neuroimaging and biological samples collected during gestation or from individuals with NDDs, may elucidate how long-term dysregulation of the immune system disrupts the development of important functional and/or structural brain regions and neural circuits in offspring. However, these methodologies cannot determine much about the cellular or molecular contributions toward NDDs, which are likely the first systems to be disrupted by an environmental or biological risk factor.

Furthermore, biological sex or possibly gender are likely critical factors that must be considered when examining the etiology of NDDs and their symptoms. Males are, on average, twice as likely than females to be diagnosed with developmental disorders, including ADHD, ASD, schizophrenia, and general learning disabilities (Polyak et al., 2015; Pinares-Garcia et al., 2018). Many of the epidemiological studies that are reviewed above did not include sex or gender as a factor in the study design nor statistical analysis. Although there are a few studies reviewed by Ardalan et al. (2019) that describe sex differences in cytokine expression of individuals diagnosed with ASD, their findings are not specifically related to MIA. For instance, males had a significantly higher risk for ASD in a Lebanese case–control study (Guisso et al., 2018) and for schizophrenia in a Finnish cohort study (Jones et al., 1998), independent of prenatal exposure to infection. There is one Boston case–control study in the United States that examined the association between cytokine levels in maternal serum collected throughout pregnancy and risk for schizophrenia in offspring (Goldstein et al., 2014). The researchers found a significant interaction between sex and subject group, such that females with schizophrenia were more likely to have decreased levels of maternal TNF-α as compared to males with schizophrenia and females in the control group. Overall, there is a need for future epidemiological studies to account for sex in their experimental design in order to better understand potential interactions between sex and specific risk factors associated with NDDs. It is also important to note here that a proper experimental design including sex as a variable requires sex to be statistically included in the analysis (i.e., testing for an *interaction* between sex and another factor of interest; if there is no significant sex effect then the analysis can be collapsed across sex). Researchers should also indicate in their publications whether sex was statistically analyzed, regardless of a significant effect.

Finally, many epidemiological studies identify links between exposure to infection with a general diagnosis of ASD or schizophrenia. Other studies have instead examined the link between MIA and specific symptoms of NDDs, like in the hyperkinetic symptom of ASD study mentioned above (Liew et al., 2014). In an Australian cohort study of children with ASD, symptoms of severe social impairment were associated with reports of the mother having a history of *chronic* immune activation such as asthma or allergies (Patel et al., 2018). Similarly, a Finnish cohort study found significant associations between maternal fever during the second trimester and several behavioral outcomes in children that are characteristic of NDDs, including distress to novel situations, difficulties with task persistence and orientation, and increased social inhibition (Dombrowski et al., 2003). These studies provide excellent examples of how we can tailor future human studies and animal models of MIA to the RDoC and examine specific behaviors that are implicated in many NDDs. The findings of these epidemiological studies can help to advance the field of neuroscience by providing the opportunity to bring rodent model research into better alignment with our current understanding of human NDDs and the associated presentation of their symptoms.

In all, the epidemiological studies are unable to show strong causal associations between any one particular infection type and a specific NDD outcome. The only possible exception being rubella, which produces its own congenital syndrome that mirrors or includes many of the symptoms of other NDDs. Furthermore, the epidemiological data do not always provide the most consistent results regarding the association between MIA and NDDs. It is possible that the relationship between prenatal infection and NDDs in studies with null findings may be clouded by other factors related to the infection or sickness response—such as fever, severity of infection, gestational timing, medication or treatment, genetic predisposition, individual immune response, fetal sex, lifestyle, etc.—that aren’t always measured or accounted for in epidemiological studies. These factors may have a significant moderating effect in whether overt MIA results in the onset of NDDs later in life (Flinkkilä et al., 2016). That said, the majority of epidemiological evidence does support the hypothesis that MIA during gestation, in and of itself, is a risk factor for various NDDs, rather than being due to the unique biological characteristics of any one pathogen or immunogen.

## 4. The maternal immune system during pregnancy: Cytokines in the absence of infection

The immune system functions under very tight regulation, such that immune activation cannot be too robust but must also be sufficient to fight off infection, otherwise it can result in death. That said, there are genetic variations in the immune system and certain disorders that alter how the immune system functions across individuals. A recent example is individual differences in response to infection with the severe acute respiratory syndrome coronavirus 2 (SARS-CoV-2), also known as the COVID-19 virus. For some individuals, COVID-19 produces a robust immune response with strong cytokine production that may increase the risk of acute respiratory distress (Cummings et al., 2020), whereas others may not experience any symptoms of infection. As another example, males and females have different immune responses (Klein and Flanagan, 2016) and as a result, females are more likely to suffer from autoimmune disorders where the immune system is overactive and causes elevated cytokine levels and T-cell responses (Klein and Schwarz, 2018). The various factors that affect individual immune function can become the backdrop upon which pregnancy, and then subsequent infection, result in various outcomes in the developing fetus associated with risk for NDDs.

Many researchers in the field of developmental neuroimmunology, with a focus on MIA and NDDs, do not always account for the fact that the immune system is altered dramatically by pregnancy itself. The pregnant body goes through a process of immunosuppression in order to protect the non-self fetus from being attacked and rejected by the maternal immune system. As such, even a slight imbalance in this pregnancy-induced immunosuppression can result in early termination of the pregnancy. These changes in maternal immune function during pregnancy can allow for more severe infections to occur, particularly during late gestation, but can also temporarily alleviate autoimmune diseases for many women (Robinson and Klein, 2012; Sherer et al., 2017; Klein and Schwarz, 2018). Evidence for gestational immunosuppression has also been demonstrated in rat models. Within the 96 h before and 24 h after parturition, pregnant rats have a decreased febrile response following a *very low* dose of lipopolysaccharide (LPS; 25 μg/kg), as compared to unbred or lactating female rats. Moreover, within the 24-h period before expected time of parturition, *no* pregnant rat developed a fever and the majority became hypothermic; an effect that resulted in death in 80% of pregnant dams within 3–15 h (Martin et al., 1995). This shift in immune function is so robust that administering LPS (100 μg/kg) to a pregnant rat at embryonic day (E)11 (mid-gestation) attenuates IL-1β expression in the maternal spleen to 12%, IL-6 expression to 20%, and IFN-γ expression to 30% of the levels measured in a non-pregnant female administered the same dose of LPS (Sherer et al., 2017). By E22, the day prior to birth, the same dose of LPS produces virtually no significant cytokine production in the maternal spleen, highlighting just how dramatic the immunosuppression of pregnancy can be (Sherer et al., 2017). In the placenta and the fetal brain at E11, there is an upregulation of IL-1β and IL-6 that is modest (4-5-fold) following MIA with LPS, but then non-existent at E22, just prior to birth (Sherer et al., 2017). In conclusion, pregnancy significantly attenuates the function of the immune system, an effect that is necessary for a successful pregnancy.

In a typical healthy pregnancy, the developing fetus is exposed to very low levels of immune molecules, but there may be instances where cytokine production could become dysregulated even in the absence of infection. Fetal exposure to elevated levels of cytokines may increase the risk of NDDs and their symptoms. Supporting this theory, the human data summarized above shows that increased cytokine expression in maternal serum and/or the amniotic fluid is associated with NDDs in offspring, even in the absence of apparent, current infection. In these cases, it is possible that an underlying inflammatory condition, variations in immune function, stress-induced immune activation, or perhaps a slight shift in the typical immunosuppression associated with pregnancy may increase cytokine production and the associated risk of NDDs. This should be considered in future epidemiological and basic research.

## 5. Rodent models of immune activation to study neurodevelopmental disorders

As mentioned above, human studies are limited in their ability to establish a causal relationship between risk factors and NDD outcomes. Furthermore, it is difficult for researchers to determine the underlying neurobiology that may contribute to the disorder, as postmortem studies are limited by the number of donations and our current neuroimaging technology does not allow us to examine structural and functional neural changes at the cellular or molecular level. Therefore, animal models are necessary to understand the role of the immune system and immune activation in the perturbation of neurodevelopment.

In animal research, there are investigators that attempt to generate a model of a specific disorder (often *via* manipulation of a known genetic risk factor) and describe their research as using a rodent model of a specific NDD. However, these NDD-specific models often fail to capture the full range of symptoms and individual nuance of the disorder (Vigli et al., 2020). In recent years, researchers have begun to develop animal models that examine phenotype(s) shared across multiple disorders, rather than producing an animal model of a specific disorder. This practice is in line with the RDoC initiative from the NIMH, as described previously in Section 2. The reason for this is that symptoms of NDDs and other disorders are often overlapping, suggesting a potential for commonality in the neurobiological origins of the disorders (Conradt et al., 2021; Auerbach, 2022). MIA is often used as a solitary manipulation in animal models to examine how perturbations of the developing immune system (a risk factor for NDDs) may contribute to specific symptoms of NDDs, such as disturbances in learning, social, and sleep behaviors. Rodent models are especially important for our understanding of the neural processes that can become disrupted by MIA (particularly the inflammatory response in the maternal body and fetal compartments) and lead to NDD-associated outcomes in offspring.

However, we also know that the etiology of NDDs likely stems from a *combination* of genetic and environmental factors. Therefore, researchers have begun to utilize “two-hit” and “multi-hit” models of neurodevelopment whereby multiple inflammatory stressors (such as genetic mutations, immune challenges, diet manipulations, social stressors, etc.) are combined to examine specific phenotypes of NDDs. Utilizing genetic models of “specific disorders” that also examine other risk factors for NDDs may help us to better understand the interaction between biology and environment in the etiology of that specific disorder, rather than examining one factor by itself. Harvey and Boksa (2012) theorized that (1) the same risk factor can result in different NDDs and the characteristics of that risk factor (i.e., the dose, timing, immunogenic target, etc.) ultimately contribute to the distinct NDD phenotypes, OR (2) the same risk factor can cause different NDDs because it interacts with other vulnerability factors to contribute to the distinct NDD phenotypes. This theory should be kept in mind when considering basic biomedical research models for MIA and their associated outcomes. Next, we will discuss the various models of MIA that are commonly used by researchers in studying NDDs and evaluate their relevance to the human epidemiological data.

### 5.1. Rodent models of maternal immune activation

A range of different immunogens (any pathogens or molecules that can activate the immune system) are used in rat and mouse models to explore the underlying neurobiology implicated in the association between MIA and psychiatric phenotypes. For example, prenatal exposure to influenza in rodents produces altered expression of serotonergic and glutamatergic receptors, reduced exploration of the open arm of an elevated-plus maze (anxiety-like behavior), and deficits in prepulse inhibition (reduced sensorimotor gating; Shi et al., 2003; Moreno et al., 2011; Spini et al., 2021). Furthermore, exposure to *T. gondii* antigens during gestation in mice causes increased anxiety behaviors later in life (Webster et al., 2013; Spini et al., 2021). Exposure to diesel exhaust particles, which can also activate the immune system, during gestation and neonatal development produces learning and memory deficits in an elevated-plus and a Morris water maze, reduced social interactions, and alterations in ultrasonic vocalizations (communication deficits; Bolton et al., 2013; Chang et al., 2018; Ehsanifar et al., 2019). Further, prenatal infection with *E. coli* in rats impacts neonatal sensorimotor learning and adult spatial learning (Wallace et al., 2010). Perhaps some of the most commonly used rodent models of MIA include administration of polyinosinic:polycytidylic acid (Poly I:C) or lipopolysaccharides (LPS), which are mimetics for viral and bacterial infection, respectively.

#### 5.1.1. Poly I:C

Poly I:C is a synthetic double-stranded RNA that is used to stimulate an innate immune response through the Toll-like receptor 3 (TLR3) pathway (Reisinger et al., 2015). As described by Bao et al. (2022), Poly I:C is recognized and internalized by TLR3, which causes downstream activation of nuclear factor-κB (NF-κB), activator protein 1 (AP-1), and interferon regulatory factor 3 (IRF3). This, in turn, induces the expression of inflammatory cytokines and type 1 interferon (IFN) genes which are produced and released by the immune system in order to, respectively, stimulate an innate immune response and inhibit replication of the viral Poly I:C RNA.

Mouse and rat models of MIA report using a range of doses of Poly I:C, from 250 μg/kg to over 20 mg/kg, with doses of 4 mg/kg and 20 mg/kg being the most commonly used (Boksa, 2010; Solek et al., 2018). The gestational timing of Poly I:C administration also varies widely from embryonic days (E)9 to E19, with administration most commonly reported on E12.5 and E15. Some labs also report using multiple exposures Poly I:C, such as across two consecutive days of gestation or on two different days such as E9 and E17 (Boksa, 2010; Solek et al., 2018).

#### 5.1.2. Lipopolysaccharide

LPS is a cell-wall component of gram-negative bacteria (like *Escherichia coli*) that can also be used to stimulate an innate immune response *via* the TLR4 pathway (Reisinger et al., 2015). With the help of cluster of differentiation 14 (CD14), LPS binds to proteins on TLR4 which, similarly to Poly I:C, causes downstream gene expression and production of inflammatory cytokines and type 1 interferons (IFN) (Bae et al., 2010; Bao et al., 2022). Although interferons (particularly IFN-ɣ) are vital for the immune response to viruses, the activity of IFN-α and INF-β can also help the immune system fight off other pathogens such as bacteria (Bae et al., 2010).

Researchers report using dosages of LPS ranging from 25 μg/kg to over 1 mg/kg, with 100 μg/kg being more commonly used (Boksa, 2010; Solek et al., 2018). The timing of LPS administration varies across gestation, from E9 to E19, with administration most commonly reported around E15 and E18. Some models also employ multiple hits of LPS, such as subsequent injections on E15 and E16 or on E18 and E19. The utility of multiple hits of LPS is lessened by evidence that the peripheral cytokine response is attenuated after one previous LPS exposure (Wendeln et al., 2018). However, there is evidence to suggest the neural cytokine response is *augmented* to a second, but not a third or fourth, “hit” of LPS (Wendeln et al., 2018). Though, in the context of MIA, the maternal peripheral immune system is what influences the cytokine response in the fetal compartment; there is no evidence to support that the maternal *neural* immune response is implicated in the association between MIA and NDDs. Therefore, it is unclear how multiple hits of LPS during gestation would affect the developing fetal brain any differently than a single hit of LPS.

#### 5.1.3. Relevance of rodent models to human studies of maternal immune activation

Several review articles have compiled data from rat and mouse MIA models that examine behavioral and neural outcomes following exposure to prenatal Poly I:C and LPS (see Meyer et al., 2009; Boksa, 2010; Solek et al., 2018; Kentner et al., 2019; Bauman and Van de Water, 2020). Some of the behavioral outcomes of rodent MIA studies that are summarized in these reviews include: deficits of latent inhibition and prepulse inhibition, reduced open-field exploration and novel object recognition, decreased social interactions, altered spatial memory in novel object location and Morris water maze tasks, and increased repetitive behaviors such as grooming or stereotyped behaviors in an open field. These rodent behavioral phenotypes are relevant to humans in that they align with the common overlapping symptoms of NDDs, such as deficits in sensorimotor gating, learning disabilities, and altered social behaviors, as we previously described. Such findings support the RDoC framework. Further, these data highlight how maternal inflammation during pregnancy, even in the absence of overt infection, can produce NDD-associated behavioral changes in offspring, likely through the disruption of neural processes necessary for brain development.

Similar to the human epidemiological data summarized above, the severity of the immune activation—related to immunogen dose in rodent models—may be implicated in neurodevelopmental outcomes related to NDDs. MIA with Poly I:C, and with lower doses of LPS (100–500 μg/kg), can cause subtle neural changes such as long-term alterations in cytokine expression, changes in neurotransmission, reduced proliferation of new neurons, and changes in microglia activation measured in adolescent and/or adult offspring (see Boksa, 2010; Solek et al., 2018; Kentner et al., 2019; Hameete et al., 2021 for more detailed information). On the other hand, larger doses of LPS (more than 1 mg/kg) administered during gestation can produce severe damage to white matter, axons, and dendrites (Fan et al., 2005). These MIA-driven neural changes may occur during fetal development but are often prolonged and measurable throughout offspring postnatal development and into adulthood. Again, many of these neural changes are thought to be driven by the maternal response to MIA, which can interact with the fetal compartment and disrupt immune molecules and immune cells like microglia from performing their essential functions during neural development (i.e., synaptic pruning, neural circuit formation, etc.). Moreover, sex differences have been explored in rodent models of MIA and are detailed in other review articles (Ardalan et al., 2019; Bauman and Van de Water, 2020; Breach and Lenz, 2022), with the takeaway being that MIA differently affects the behavioral and neural phenotypes of male and female offspring. Below, we will further discuss the importance of offspring sex when studying the association between MIA and NDDs.

Taken together, rodent models of Poly I:C and LPS, which mimic viral and bacterial infections, respectively, seem to be effective in modeling the behavioral phenotypes of NDDs, particularly ASD, schizophrenia, and generalized learning deficits. Rather than employing a direct infection, Poly I:C and LPS are typically administered either intraperitoneally or subcutaneously, thereby stimulating the innate immune system in a similar manner to a peripheral infection in humans, but without the full infection (Ashdown et al., 2006). This is particularly relevant given the epidemiological data summarized above, which suggest that the maternal immune response (including fever, cytokine production, altered fetal microglia function, etc.) may precipitate NDDs in offspring rather than the direct infection itself. Interestingly, neonatal administration of LPS actually produces a broader and more robust neuroimmune response in rat hippocampus than *E. coli* (Schwarz and Bilbo, 2011a). Similarly, Poly I:C is more likely to result in activation of the rodent immune system, given that many common human viruses are not pathogenic in rodents. Though, many human viruses can be adapted for use in a rodent model if part of the immune system (e.g., IFN-ɣ) is knocked out of the rodent genome (Brehm et al., 2013; Sarkar and Heise, 2019). Thus, LPS and Poly I:C in rodent models may allow us to characterize the effects of MIA on neurobiological processes underlying symptoms of NDDs, independent of the infection itself.

One major limitation in our ability to interpret various rodent models of MIA is the variability in experimental design across labs, including: gestational timing and frequency of the immune challenge, serotype/strain/dose of the immune challenge, age at which behavioral measures are tested in offspring, the types of behaviors and neural outcomes measured within a lab, and mouse versus rat models (whose immune systems are quite distinct). More specifically, researchers should determine whether their model of MIA may be better studied in mice versus rats, depending on the intended experimental manipulation and measured outcomes. For instance, mouse models are currently better suited than rat models for manipulations whereby researchers can examine the role of particular genes in NDD risk. On the other hand, rats are often more adept at performing complex learning and behavioral tasks, deficits in which may be associated with NDDs (Parker et al., 2014). Furthermore, researchers should consider that different strains of mice and rats often display distinct biological and behavioral profiles, and that this can even be influenced by the vendor from which the animals are sourced (Kentner et al., 2019). Nonetheless, both mouse and rat models of MIA seem to yield similar patterns of behavioral and neural findings related to NDD risk and symptomatology, even with varying rodent strains, offspring age at the time of measured outcomes, and dosages/gestational timing of the immune challenge. This provides further support that rodent models can provide insight into the neurobiological mechanisms underlying MIA-driven disruptions in offspring neural and behavioral development.

There are also inconsistencies in the reports of maternal death and fetal resorption or pup death that are often an inevitable consequence of many of these models. For example, even administration of an “ultra-low” dose of LPS (0.5 μg/kg) early in gestation (E5) results in significantly smaller litters and resorbed fetuses (Xue et al., 2015). Thus, it is likely that experiments using higher doses of LPS or PolyI:C produce a much more severe immune activation in the dam and fetuses. The question then remains whether this associated fetal mortality is actually modeling simple maternal infection (MIA related to NDDs), or rather modeling more severe infections like chorioamnionitis or endometritis that are associated with serious birth complications like premature birth or stalled labor. On the other hand, one potential benefit of these experimental inconsistencies is that they mirror the in outcomes variability in outcomes that can occur within the human population, even in the severity of the maternal immune response during pregnancy. In all, there are similar neurobiological and behavioral consequences reported across multiple different epidemiological and rodent studies, even with variation in the factors contributing to the models; this provides strong evidence for an association between MIA and outcomes associated with NDDs. Beyond this, researchers have also begun to utilize “two-hit” and “multi-hit” models that incorporate MIA as just one of the multiple “hits” to examine the ontogeny or symptoms of NDDs.

### 5.2. “Two-hit” and “multi-hit” rodent models of neurodevelopment

Prenatal infection is not the only risk factor for NDDs. For example, environmental stressors such as negative social interactions or social exclusion during development, particularly adolescence, have also been linked to the onset of symptoms like psychosis or aberrant processing of social cues (Li et al., 2012; Davis et al., 2016). Indeed, many environmental and psychological stressors themselves—diet, pollutants, allergens, social stress, psychological stress, depression, etc. (Carlsson et al., 2021)—are able to trigger an inflammatory state in the brain and body. These inflammatory stressors can disrupt neural and behavioral development in offspring, both when experienced fetally during MIA and/or postnatally by the offspring. As such, the “two-hit” and “multi-hit” hypotheses of NDDs suggest that a *combination* of environmental, psychological social, or genetic “hits” throughout development significantly increases the overall risk for an individual to be diagnosed with NDDs such as ASD and schizophrenia (Davis et al., 2016), in addition to many comorbid disorders including general anxiety, depressive symptoms and learning disorders. Some risk factors associated with NDDs that have been examined in Poly I:C mouse models of MIA include genetic models of DISC1 (Disrupted in Schizophrenia 1) mutation, acute stress during juvenile development, and pubertal social isolation (Yee et al., 2011; Solek et al., 2018; Goh, 2020). Researchers should continue to develop more complex models of MIA that incorporate various other risk factors, in order to better understand how environmental and genetic factors mediate individual differences in the maternal and fetal immune responses and drive alterations in the behavioral and neurobiological development of offspring.

## 6. Factors to consider in rodent models of maternal immune activation

There are many factors that should be considered by researchers when studying NDDs, as they have an important role in the etiology and/or manifestation of symptoms of NDDs. These factors include (1) developmental timing of the immune challenge, (2) sex of the offspring, and (3) individual factors that may influence one’s immune response, such as genetics, parental age, the gut microbiome, prenatal stress, and placental buffering.

### 6.1. Developmental timing of the immune challenge

The gestational timing of MIA may affect the fetal and maternal immune response differently throughout pregnancy. Individual differences in the immune response may be influenced by genetic predisposition to the infectious agent (Carter, 2009) and by the influence of pregnancy itself on the immune system (Sherer et al., 2017), as we described earlier. Furthermore, in both human and rodent studies, there is evidence that the timing of MIA can alter behavioral and neural outcomes in both the mother and offspring. For instance, maternal estradiol levels are lower during early stages of pregnancy compared to late stages, and ERβs begin to be expressed in fetal tissues around 16–18 weeks of gestation (Takeyama et al., 2001; Shepherd et al., 2021). Incubation with estradiol decreased levels of LPS-induced TNF and IL-6 cytokine production in infant cord blood mononuclear cells (Giannoni et al., 2011), suggesting that the circulating pregnancy hormones from the mother may impact the fetal immune response, in addition to the maternal immune response, as we already described. As another example, maternal infection with Zika virus during the first half of pregnancy is associated with greater rates of birth defects than during the latter half of pregnancy, likely due to the targeting of proliferative cells in the early developing brain (Honein, et al., 2017; Pomar et al., 2017).

The timing of MIA matters because it may affect different neurodevelopmental processes occurring during fetal development at that time. It is important to keep in mind that the gestational timing of animal models is shifted relative to that of humans, whereby the gestational period of mice is generally 21 days, of rats is 23 days, and of humans is 40 weeks. The first and second halves of gestation in rodents is approximately the equivalent of the first and second trimesters in humans, whereas the human equivalent to the third trimester in rodents occurs during the first 2 weeks of neonatal life, because rodent pups are born altricial. Therefore, many important neurodevelopmental processes—neurogenesis, immunogenesis, apoptosis, synaptogenesis—occur during gestation in humans but continue post-birth in rodents (Estes and McAllister, 2016; Guma et al., 2019). Moreover, some of these neurodevelopmental processes may be affected differently if the immune response occurs during early stages versus later stages of the developmental process (Bauman and Van de Water, 2020).

Overall, future research should take a more systematic approach to evaluate the effects of gestational timing within rodent models of MIA and try to better characterize which neurobiological processes are being studied and thus perturbed during fetal vs. postnatal neurodevelopment. In doing so, we should keep in mind that the later fetal developmental processes in humans are still being modulated by maternal biology, hormone production, and immune responses, whereas neurodevelopment in rodents continues postnatally, without these influences.

### 6.2. Sex of the offspring

As mentioned previously, NDDs—such as ASD, ADHD, and early-onset schizophrenia—are more commonly diagnosed in males than in females. There may be two reasons for such discrepancies: (1) the manifestation of symptoms in females is different than in males, and the current diagnostic criteria is more aligned with symptoms commonly presented in males, and/or (2) the disruption of neurobiological processes that cause NDDs are more likely to occur in males than in females. It is also possible that certain neural and behavioral processes mature at different rates between males and females, and therefore exposure to immunogens may differentially affect males and females depending on the developmental timing of the exposure. Despite the known sex-bias, there are limited epidemiological data investigating how sex may impact the role of MIA as a risk factor for NDDs, because research does not always seek out an equal female-matched comparison group (D’Mello, 2022). Moreover, rodent models themselves can contain sex biases in experimental design and analysis of results. For instance, many behavioral protocols were generated and validated when the use of only males in rodent studies was common (Beery and Zucker, 2011; Shansky and Murphy, 2021), which makes it difficult to assess the same behavioral endpoints in females. Studies that do now include the use of both male and female subjects often lack substantial power to statistically detect sex differences, or fail to examine the data for sex differences at all (Coiro and Pollak, 2019). Nevertheless, when properly designed to account for potential sex differences, rodent models can help us identify how MIA may impact the neurodevelopment of males and females differently and contribute to the variety of phenotypes relevant for NDDs. Sex differences have been successfully explored in rodent models of MIA and are detailed in other review articles (Ardalan et al., 2019; Bauman and Van de Water, 2020; Breach and Lenz, 2022).

One mechanism by which sex of the offspring may interact with MIA may be through estradiol receptors (ERα and ERβ). Estradiol regulates the activation of innate immune signaling pathways and can influence the synthesis of pro- and anti-inflammatory cytokines by the NF-κB pathway (Kovats, 2015; Liu et al., 2017). For example, estradiol (E2) can inhibit this pathway *via* increased production of IκBα mRNA (Xing et al., 2012). The expression and activation of ERs vary between males and females, which causes differences in the magnitude and duration of the innate inflammatory response between sexes (Kovats, 2015; Arnold and Saijo, 2021). For instance, females have a higher basal expression of ERα and ERβ than males in human blood monocytes-derived macrophages (MDMs) (Campesi et al., 2017). Similarly, female mice had a higher basal density of ERβ relative to male mice at postnatal day 21 (P21) in the anteroventral periventricular nucleus (AVPV), an area important for cardiovascular functions supporting female reproduction (Zuloaga et al., 2014; Saper and Stornetta, 2015). When human MDMs were incubated with 100 ng/ml of LPS for 24 h, both expression and phosphorylation ERα were upregulated to a larger degree in males than females (Campesi et al., 2017). Thus, it is possible that the female immune response to pathogens may occur on a temporally faster timeline than males, due to a lesser need to express or phosphorylate ERs in response to the immunogenic insult.

Sex differences in the density, maturation, or activation of microglia, the innate immune cells of the brain, may also contribute to variability in immune response between males and females (Schwarz et al., 2012; Klein and Flanagan, 2016; Hanamsagar et al., 2017; Ardalan et al., 2019). As discussed above, microglia have an active role in the developmental pruning and maturation of synapses, and compromising these functions can lead to alterations in neural circuit development and deficits in learning (Paolicelli et al., 2011; Schafer et al., 2012; Zhan et al., 2014; Tay et al., 2017). One hypothesis may be that MIA alters the number or activational state of microglia differently in males and females, which may contribute to sex differences in the ontology and manifestation of various NDDs. Indeed, microglia with a stout and ameboid morphology—which commonly occur when microglia are activated during an immune response or insult—are more prevalent in females than males from P0–P4 and from P30–P60 (Schwarz et al., 2012; Hui et al., 2020). Differences in microglial activation state may potentiate differences in the neuroimmune response between males and females (Osborne et al., 2018). The role of microglia in the maternal neuroimmune response to MIA has been well-studied, however there are often conflicting findings due to differences in study design and analysis methods (see review: Smolders et al., 2018).

Overall, there is a need to examine sex as a factor in both human and rodent studies of MIA. It is important to mention there are sex biases in the experimental designs, the inclusion of male and female animal subjects, and the neurochemical analyses of MIA studies (Coiro and Pollak, 2019). It is again essential to note that a proper experimental design including sex as a variable requires sex to be statistically included in the analysis (i.e., testing for an interaction between sex and another factor of interest). Researchers should also report that sex was included in their analyses, even when there are no significant findings. In all, developing well-designed experiments that include sex as a variable can help us better identify how neurobiological processes are differently dysregulated by the maternal and fetal immune response and how sex may interact with MIA to contribute to differences in NDD diagnostic rates between males and females.

### 6.3. Individual differences in immune response

There is often high variability in the maternal and fetal immune response when examining cytokine expression in rodent models of MIA, which suggests that there may be individual differences in the immune response to MIA (Sherer et al., 2017). This individual susceptibility or resilience to MIA can also manifest in offspring behavioral outcomes. For example, one study found that MIA with Poly I:C resulted in two groups of adult offspring with distinct behavioral phenotypes: those with enhanced prepulse inhibition (PPI) and those with deficits in PPI, as compared to saline-exposed offspring (Chamera et al., 2021). Interestingly, only the MIA-exposed offspring with enhanced PPI had altered protein levels of CX3CL1-CX3CR1 (molecules involved in microglia–neuron signaling, important for synaptic organization) in the frontal cortex and hippocampus. It is also essential for researchers to consider how differences in the immune response at the litter level (maternal immune response) or at the offspring level (fetal or postnatal immune response) may impact their experimental and/or statistical design. A few articles (see Lazic and Essioux, 2013; Weber-Stadlbauer and Meyer, 2019) have been published to help guide researchers experimentally and statistically account for sources of variability in rodent models.

Individual susceptibility or resilience in the response to MIA, at both the maternal and fetal levels, indicate that other biological, environmental, and genetic factors may have an influence on offspring outcomes related to NDDs (Meyer, 2019; Herrero et al., 2023). It is also possible that these other factors—such as genetics, parental age, dietary deficiencies, stress, and placental buffering—may contribute to or account for some of the observed immune and behavioral variability in human and rodent studies of MIA and NDDs. It is therefore essential to take such factors into consideration when designing rodent and human studies of MIA and, rather than shy away from potential variability within the data, investigate the potential factors that may have individual or multiplicative effects on MIA and subsequent predisposition to NDDs.

#### 6.3.1. Genetics

Twin studies have identified a high concordance among monozygotic (MZ) twins that is much lower in dizygotic (DZ) twins, demonstrating that many NDDs—namely ASD or schizophrenia—have a strong genetic link (Tick et al., 2016). That said, while thousands of genes, copy number variants, and *de novo* mutations have been associated with NDDs, to date there have been no risk loci identified that are common within each type of NDD or across all types of NDDs (Vorstman et al., 2017; López-Rivera et al., 2020). Rather, epidemiological data suggest that genetic risk provides a foundation upon which other factors may precipitate or enhance the risk for many NDDs (Zawadzka et al., 2021). It is possible that this genetic risk could be hereditary in nature, as both a familial history of psychiatric disorders and a parental history of severe infections seem to be involved in the association between MIA and offspring NDD risk, as discussed above.

Accordingly, genes that are implicated in schizophrenia may also impact how the body processes and fights off different pathogens, including influenza, rubella, and *T. gondii* (Carter, 2009), which suggests that individuals with these genes may be more prone to infections and, in turn, more at risk of NDDs as a consequence of the maternal infection. Similarly, people with ASD have an upregulation in genes that regulate neural cell development, but also in genes that regulate the immune response, the inflammatory response, antigen production and presentation, as well as immune cell signaling (Voineagu et al., 2011; Voineagu and Eapen, 2013). These genetic markers can also increase one’s susceptibility to other inflammation-inducing factors, such as diet, physical stress, psychological stress, etc. There is also evidence that MIA produces transcriptional changes in expression of inflammatory markers, GABAergic signaling proteins, and myelin, and may drive epigenetic changes in the transcription of genes associated with NDDs (Woods et al., 2021). For example, MIA with Poly I:C in mice produced an integrated stress response (ISR) in male offspring, associated with increased phosphorylation of eIF2α which is important for cellular translation (Kalish et al., 2021). Therefore, genetic influences may impact both the maternal immune response as well as the neurodevelopmental and behavioral processes in offspring that are ultimately affected by the immune response.

#### 6.3.2. Parental age

Maternal and paternal age have also been implicated in the risk for certain NDDs. For instance, older maternal and paternal ages have been linked with increased risk for ASD (Abdallah et al., 2012; Sandin et al., 2012; Carlsson et al., 2021). Advanced paternal and maternal ages have also been associated with increased risk for schizophrenia and psychosis (El-Saadi et al., 2004; Lopez-Castroman et al., 2010; Fountoulakis et al., 2018). On the other hand, younger maternal age has also been implicated in risk for psychosis, when controlling for paternal age (El-Saadi et al., 2004). Younger maternal and paternal ages have been associated with increased risk for ADHD diagnosis overall (Chang et al., 2014; Hvolgaard Mikkelsen et al., 2017). Interestingly, older maternal age and younger paternal age have been associated with hyperactivity/impulsivity symptoms of ADHD, whereas younger maternal and paternal ages have been linked to inattentive symptoms of ADHD (Ghanizadeh, 2014; Sciberras et al., 2017).

Not much is known about *how* parental age influences the risk of NDD in children. Maternal age may influence the immune response to MIA, given that immune function changes with age (though usually much older ages; see Haynes, 2020). Increased maternal age is also associated with an increased risk of pregnancy and obstetric or birth complications that are often associated with inflammation, such as preeclampsia, gestational diabetes, or general hypertension (Londero et al., 2019). The role of paternal age as a risk for NDDs implies that genetics may also have a role in this relationship. As the body ages, there is a greater risk for genetic mutations in the eggs or the sperm that would contribute to an increased risk of NDDs. Moreover, environmental exposure to toxins or infections throughout the lifespan may also result in *de novo* genetic mutations that can be passed to offspring and increase their risk for NDDs, an effect that appears to happen more frequently in sperm than in eggs (Kong et al., 2012; Jónsson et al., 2017).

Notably, the age of the dam or sire are not consistently reported or controlled for in animal models, nor how many litters that any one dam has had previously. Furthermore, there are few to no animal studies that have examined maternal or paternal age as a risk factor that may interact with MIA or developmental outcomes in the offspring. As with all controlled rodent studies, reporting the age of the mating pair is important, whether or not it has an effect on MIA or on the behavioral outcomes in the phenotype being examined. Human epidemiological studies often control for age as a covariate when examining the link between MIA and NDDs, however few studies actively include it as a variable in their overall analysis. As future studies characterize the role of aging in the risk of NDDs, they should consider use of a multivariate model that considers parental age, along with infection during or before pregnancy in either the mothers or the fathers, to get a better understanding of how these risk factors interact.

#### 6.3.3. The gut microbiome and dietary factors

The gut microbiota can be impacted by diet and by metabolic conditions. Maternal metabolic conditions such as obesity, diabetes, and hypertension have been associated with increased risk for ASD (Van Lieshout and Voruganti, 2008; Krakowiak et al., 2012). In mice, maternal high-fat diet has been shown to produce microglia-associated changes in myelination and increase the number of perivascular microglia in the offspring brain (Bordeleau et al., 2021, 2022), as well as cause offspring to have less diverse gut communities, decreased oxytocin production in the paraventricular nucleus of the hypothalamus, and diminished synaptic plasticity in the ventral tegmental area (Buffington et al., 2016). Maternal high-fat diet itself has been used to model MIA in rodents, as it can trigger a chronic inflammatory profile in the dam and can produce behavioral phenotypes in offspring that are related to NDDs, including increased repetitive behaviors and disruptions in social and cognitive behaviors (Sullivan et al., 2015; Buffington et al., 2016; Penna et al., 2020; Bordeleau et al., 2021, 2022). Gestational diabetes may also interact with MIA to impact neurodevelopmental processes in offspring (Van Lieshout and Voruganti, 2008). Prenatal exposure to both gestational diabetes mellitus and Poly I:C in mice resulted in offspring with an altered transcriptional profile of genes that are associated with differentiation of dopamine neurons and the innate immune response (Money et al., 2018). Finally, antibiotic use can also alter the composition of the gut microbiota (Patrono et al., 2021). In humans, second trimester influenza infection associated with ASD risk was only apparent when antibiotics were *not* taken at any point during the pregnancy (Holingue et al., 2020), which suggests that the antibiotics may have altered the microbiome in a way that prevented the negative consequences of influenza from affecting the developing fetal brain.

The gut microbiota are essential in regulating the immune system, including the proliferation and differentiation of T- and B-cells that drive the maternal cytokine production implicated in MIA (Minakova and Warner, 2018). Certain forms of commensal gut bacteria, like segmented filamentous bacteria, are more likely to induce differentiation of T-cells that produce IL-17a, which is a cytokine that has consistently been associated with behavioral changes (particularly decreased social behaviors) and cortical abnormalities in various models of MIA (Kim et al., 2017). Colonization of Pregnant female mice that were colonized with segmented filamentous bacteria, then challenged with Poly I:C on E12.5, were more likely to produce TH-17 cells and have offspring with distinct behavioral phenotypes characteristics of NDDs (Kim et al., 2017), likely triggered by exposure to the enhanced IL-7 production from the maternal gut’s adaptive immune cells (Kim et al., 2017). Similarly, increased levels of pro-inflammatory cytokines in the gut have also been associated with the positive symptoms of schizophrenia in humans (Patrono et al., 2021). Taken together, the gut microbiome may prove useful in providing additional biomarkers for immune dysregulation associated with NDDs or as targets for therapies against NDDs, particularly if the microbiome changes in concert with, or before the onset of, symptoms for many NDDs.

Maternal diet deficiencies of iron, omega-3 fatty acids, and folic acid may also impact neurodevelopmental outcomes in rodents and humans in the context of MIA. Long ago, researchers determined that folic acid was necessary as part of the maternal diet to ensure proper development of the fetal neural tube (Greenberg et al., 2011). In mice exposed to LPS on E17, omega-3 deficiency in the maternal diet caused increased IL-6 expression in maternal plasma, placenta, and fetal brain (Richardson and Ross, 2000; Labrousse et al., 2018). Adult offspring exposed to both the MIA and omega-3 deficiency during development had spatial memory deficits in a Y-maze task. Furthermore, in humans, anemia, with or without exposure to prenatal infection, is associated with an increased risk for schizophrenia (Nielsen et al., 2016). In rats, dams fed an iron-deficient diet had increased serum levels of IL-6 and TNF-α following prenatal LPS on E15, as compared to typical chow-fed dams (Harvey and Boksa, 2014). Moreover, exposure to iron-deficiency and to MIA independently caused deficits in the offspring’s development of various sensorimotor behaviors. In all, there is limited evidence of multiplicative effects between dietary iron deficiencies and MIA exposure, however both seem to be independently implicated in the risk for NDDs.

Perhaps with growing evidence such as that described here, future research should examine whether the gestational/developmental timing of dietary deficiencies or alterations in the gut microbiota may interact with MIA to increase the risk of NDDs. In turn, studies should examine whether diet-derived supplementations might mitigate the effects of MIA. For example, maternal dietary supplementation with choline, around the time of birth in rats, attenuated the splenic cytokine immune response of 3-week-old offspring to an *ex vivo* immune challenge (Richard et al., 2017). In addition, several dietary factors—including high maternal iron, zinc, and vitamin D—have been associated with resilience to effects of MIA *via* anti-inflammatory cytokine production and enhancement of antioxidant systems (Vuillermot et al., 2017; Meyer, 2019).

#### 6.3.4. Prenatal stress and inflammation

Prenatal stress has long been associated with an increased risk of various NDDs, most notably schizophrenia, ADHD and autism (Ronald et al., 2011; Diz-Chaves et al., 2012, 2013; Chan et al., 2018; Minakova and Warner, 2018; Makris et al., 2022). More recently, this association has been further characterized by changes in inflammatory biomarkers in the maternal circulation that may increase the risk of various NDDs. For example, even socioeconomic disadvantage is a stressor that is associated with transcriptional indications of greater immune activation and slower tissue maturation in the placenta (Miller et al., 2017). This stress can lead to overproduction of pro-inflammatory cytokines by immune cells in response to additional immunostimulation (Miller et al., 2017). Stress-induced susceptibility to MIA may be linked to changes in baseline maternal cortisol levels (Van den Bergh et al., 2005), resulting in continuously elevated or stimulated pro-inflammatory cytokine levels that may impact fetal neurodevelopment associated with NDD risk.

Animal models of prenatal stress have demonstrated a pro-inflammatory cytokine response, particularly IL-6, with microglial activation similar to that elicited by MIA models. Specifically, prenatal stress in rodents enhanced cytokine levels in the hippocampus and increased the total number of immunoreactive microglial cells in the offspring compared to non-stressed animals, which exacerbated the inflammatory response to LPS (Diz-Chaves, et al., 2012). Behavioral phenotypes of anxiety, learning deficits, and depressive-like symptoms in prenatally stressed rat and non-human primate offspring are further associated with maternal and fetal HPA-axis alterations (Weinstock, 2005; Weinstock, 2008). Gestational stress and excess corticosterone in maternal and fetal plasma can impair feedback regulation of the HPA axis in both infancy and adulthood and can increase corticotropin-releasing hormone (CRH) activity in the amygdala (Van den Bergh et al., 2005; Weinstock, 2005; Weinstock, 2008). Excess amounts of CRH and cortisol that reach the fetal brain during periods of chronic maternal stress could thereby influence how the fetal brain responds in the presence of MIA, or how the brain is programmed to respond to subsequent stressors or immune challenges later in life.

Animal models often fail to report or account for unintended stressors in their models that may interact with MIA to exacerbate the neural and behavioral consequences in dams and offspring. For instance, stress associated with ambient noise levels, bedding levels, handling, injection procedures, behavioral tests, caging conditions, and nearby construction are all factors that may commonly occur throughout the course of an experiment. Researchers should take care to reduce exogenous stressors wherever possible, and when unable to control for such factors, should document and report them in the literature.

#### 6.3.5. The placenta: Protector or instigator?

The placenta is an important organ that connects mother and fetus, providing oxygen and nutrition to the baby while protecting the delicate fetus from certain factors, most notably infections, that could harm it. That said, while it is well-known that many pathogens and larger immunogenic molecules *do not* cross the placental barrier, the placenta might also be implicated in the active transfer of immune molecules through the circulation to the fetus (Robbins and Bakardjiev, 2012). Unfortunately, research examining the site of the placental transfer of cytokines associated with MIA is sparse. Nevertheless, it is important to understand the role of the placenta as a site of cytokine transfer during MIA.

Decades ago, research indicated that monozygotic twins concordant for schizophrenia were more likely to have been monochorionic and to have shared a single placenta, whereas discordant monozygotic twins appear more likely to have been dichorionic with separate placentas (Davis et al., 1995). In human twin pregnancies with a conjoined placenta, the dividing membrane between the two placentae can be composed of four layers—the amnion and chorion of each twin—which allows some degree of shared circulation between the two fetuses (Benirschke, 1990). In this case, each twin may be exposed to similar circulating molecules, such as cytokines, from the mother. Maternal immune and endothelial cells come into contact with extravillous fetal cells at the uterine implantation site, allowing for maternal blood to surround the epithelial covering of placental cells, called syncytiotrophoblasts.

The syncytiotrophoblasts have been shown to be resistant to infections and thereby may contribute to the protective function of the placenta. At the same time, they are a type of immune cell that can initiate their own cytokine response in the presence of innate immune receptor activation. Due to their hemochorial nature, the placental buffer in rats and humans function in similar ways. Like in humans, the trophoblast epithelium of the rat placenta is directly bathed in maternal blood (Furukawa et al., 2019). In humans, this occurs at the decidua, the site of uterine implantation, which only has one dividing layer (the syncytiotrophoblasts). However, the rat has three layers at this site, which might imply differences in the fetal-maternal exchange processes between the two species. In both species, uterine natural killer (NK) cells are present in parts of the placenta, and help the uterus to adapt and accommodate for the fetus. In rats, MIA with Poly I:C can increase maternally-derived IL-6 protein directly in the placenta, which activates the JAK/STAT3 pathway and causes expression of acute phase immune genes in the placenta that can enter into the fetal circulation (Hsiao and Patterson, 2011). In humans, IL-6 is transferred bidirectionally between maternal and fetal circulation (Zaretsky et al., 2004). While studies suggest that many immunogenic molecules, like LPS, do not cross the placental barrier (Ashdown et al., 2006; Ning et al., 2008), there remains some debate of whether fetal immune activation by way of MIA occurs *via* the reception of cytokines from the maternal circulation or *via* an immune response precipitated in the placenta itself. Thus, additional research should be performed to examine the rat placental barrier and its potential ability to transfer immune molecules from maternal circulation.

Moreover, early work has identified dichorionic monozygotic twins as having a *lower* rate of concordance for various NDDs (Davis et al., 1995), which should also be considered within the context of rodent pregnancies where each pup has its own placenta and might respond differently to MIA. Hormones can travel through the multiple placentae among fetuses due to the blood flow of the mother. More specifically, in pregnant rats, blood flows from the caudal to distal direction, or from cervix to ovaries. Thus, a rat fetus located at the cervical end of the uterus will receive maternal blood flow prior to fetuses in other uterine positions. In litter-bearing mammals that have multiple pregnancies, effects of intrauterine position on fetal development have been observed (Ryan and Vandenbergh, 2002). For instance, female fetuses that develop downstream from male fetuses have been shown to exhibit slightly masculinized anatomical, physiological, and behavioral characteristics as adults, including altered hormone levels and disrupted endocrine systems (Ryan and Vandenbergh, 2002). This is due to diffusion of testosterone from male fetuses to their uterine neighbors *via* amniotic fluid and the maternal circulation. Given this mechanism of hormonal transfer, it may be possible for the same type of transfer to occur with immune factors such as cytokines; this may result in differential exposure to MIA-associated molecules between fetuses based on their uterine position. It is also possible that some fetuses may be more exposed to the circulating maternal immune molecules from the pregnant dam in MIA models, particularly those located more caudally as they are the first to receive maternal blood flow.

In all, more research is needed to consider the role of the placenta in the fetal response and susceptibility to the inflammatory effects of MIA. Researchers should consider how differences in rodent and human pregnancies—particularly the number of fetuses, characteristics of the placental barrier, and maternal transference or fetal production of cytokines and other immunogens—may impact the generalizability of their findings to human NDDs and the translatability of rodent models of MIA to humans.

## 7. Discussion

**Figure 1:**
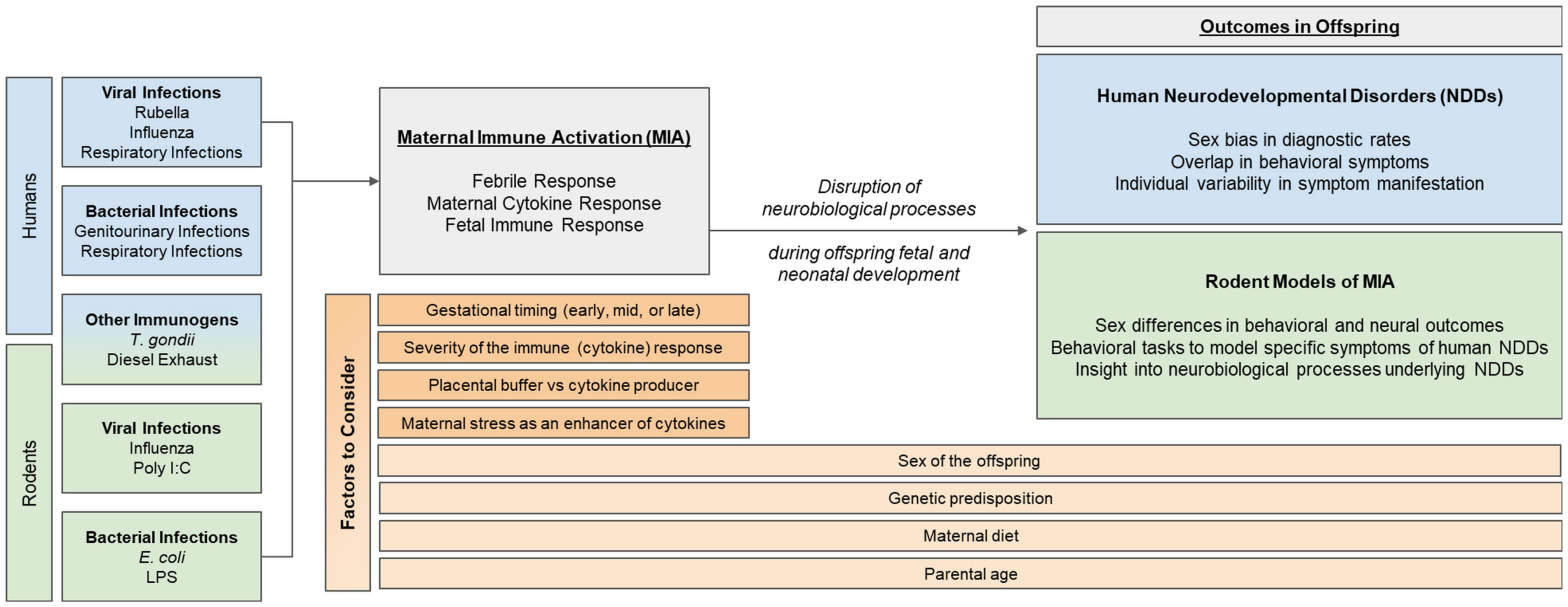
Proposed model of the association between maternal immune activation (MIA) and neurodevelopmental disorders (NDDs). Blue boxes represent human pathogens and outcomes related to MIA. Green boxes represent rodent immunogens and outcomes related to MIA. Orange boxes represent factors that should be considered in rodent models of MIA and their relevance for human NDDs. Dark orange boxes represent factors that are related to the gestational immune response, whereas light orange boxes represent factors that are related to the immune response both during gestation and in postnatal offspring.

We conclude this review with a figure that identifies the various factors that may influence the developing fetus in the context of MIA and the ontogeny of NDDs (Figure 1). Our conclusion is that every study need not examine every one of these factors in their experimental design. Rather, basic research that investigates the effect of MIA on NDDs should consider these factors when analyzing and interpreting their data. Kentner et al. (2019) have introduced a list of reporting guidelines for animal models of MIA in an effort to help standardize MIA models, to provide transparency in variability of these factors across labs, and to better enable reproducibility of findings across laboratories. Studies may still contain variability associated with these factors of consideration that we have introduced; however, this variability is similar to that observed in the risk factors and behavioral symptoms associated with human NDDs. Further investigation is still required for us to better understand the general effects of each of these factors, how they interact with perinatal immune activation (particularly with regard to the degree and severity of the MIA response), and how they contribute to the ensuing manifestation or ontogeny of the behavioral and neural phenotypes associated with NDDs.

## Author contributions

MH, JS, DW, and ER: writing and revisions. MH and JS: editing and figure creation. All authors contributed to the article and approved the submitted version.

## Funding

This work was supported by the National Institutes of Health [NIH R01MH106553 to JS].

## Conflict of interest

The authors declare that the research was conducted in the absence of any commercial or financial relationships that could be construed as a potential conflict of interest.

## Publisher’s note

All claims expressed in this article are solely those of the authors and do not necessarily represent those of their affiliated organizations, or those of the publisher, the editors and the reviewers. Any product that may be evaluated in this article, or claim that may be made by its manufacturer, is not guaranteed or endorsed by the publisher.

## References

[ref1] Abdallah M. W. Larsen N. Grove J. Nørgaard-Pedersen B. Thorsen P. Mortensen E. L.. (2012). Amniotic fluid chemokines and autism spectrum disorders: an exploratory study utilizing a Danish historic birth cohort. Brain Behav. Immun. 26, 170–176. doi: 10.1016/j.bbi.2011.09.003, PMID: 21933705

[ref2] Abdallah M. W. Larsen N. Grove J. Nørgaard-Pedersen B. Thorsen P. Mortensen E. L.. (2013). Amniotic fluid inflammatory cytokines: potential markers of immunologic dysfunction in autism spectrum disorders. World J. Biol. Psychiatry 14, 528–538. doi: 10.3109/15622975.2011.639803, PMID: 22175527

[ref3] Albani S. H. McHail D. G. Dumas T. C. (2014). Developmental studies of the hippocampus and hippocampal-dependent behaviors: insights from interdisciplinary studies and tips for new investigators. Neurosci. Biobehav. Rev. 43, 183–190. doi: 10.1016/j.neubiorev.2014.04.009, PMID: 24769291 PMC4939092

[ref4] Al-Haddad B. J. S. Jacobsson B. Chabra S. Modzelewska D. Olson E. M. Bernier R.. (2019). Long-term risk of neuropsychiatric disease after exposure to infection in utero. JAMA Psychiat. 76, 594–602. doi: 10.1001/jamapsychiatry.2019.0029, PMID: 30840048 PMC6551852

[ref5] Allswede D. M. Yolken R. H. Buka S. L. Cannon T. D. (2020). Cytokine concentrations throughout pregnancy and risk for psychosis in adult offspring: a longitudinal case-control study. Lancet Psychiatry 7, 254–261. doi: 10.1016/S2215-0366(20)30006-7, PMID: 32035031 PMC8287973

[ref6] Ardalan M. Chumak T. Vexler Z. Mallard C. (2019). Sex-dependent effects of perinatal inflammation on the brain: implication for neuro-psychiatric disorders. Int. J. Mol. Sci. 20:2270. doi: 10.3390/ijms20092270, PMID: 31071949 PMC6539135

[ref7] Arnold M. L. Saijo K. (2021). Estrogen receptor β as a candidate regulator of sex differences in the maternal immune activation model of ASD. Front. Mol. Neurosci. 14:717411. doi: 10.3389/fnmol.2021.717411, PMID: 34531723 PMC8438209

[ref8] Ashdown H. Dumont Y. Ng M. Poole S. Boksa P. Luheshi G. N. (2006). The role of cytokines in mediating effects of prenatal infection on the fetus: implications for schizophrenia. Mol. Psychiatry 11, 47–55. doi: 10.1038/sj.mp.4001748, PMID: 16189509

[ref9] Atladóttir H. Ó. Henriksen T. B. Schendel D. E. Parner E. T. (2012). Autism after infection, febrile episodes, and antibiotic use during pregnancy: an exploratory study. Pediatrics 130, e1447–e1454. doi: 10.1542/peds.2012-1107, PMID: 23147969 PMC4451062

[ref10] Atladóttir H. Ó. Thorsen P. Østergaard L. Schendel D. E. Lemcke S. Abdallah M.. (2010). Maternal infection requiring hospitalization during pregnancy and autism spectrum disorders. J. Autism Dev. Disord. 40, 1423–1430. doi: 10.1007/s10803-010-1006-y, PMID: 20414802

[ref11] Auerbach R. P. (2022). RDoC and the developmental origins of psychiatric disorders: how did we get here and where are we going? J. Child Psychol. Psychiatry Allied Discip. 63, 377–380. doi: 10.1111/jcpp.13582, PMID: 35133013

[ref12] Avella-Garcia C. B. Julvez J. Fortuny J. Rebordosa C. García-Esteban R. Riano Galán I.. (2016). Acetaminophen use in pregnancy and neurodevelopment: attention function and autism spectrum symptoms. Int. J. Epidemiol. 45, dyw115–dyw1996. doi: 10.1093/ije/dyw115, PMID: 27353198

[ref13] Babulas V. Factor-Litvak P. Goetz R. Schaefer C. A. Brown A. S. (2006). Prenatal exposure to maternal genital and reproductive infections and adult schizophrenia. Am. J. Psychiatr. 163, 927–929. doi: 10.1176/ajp.2006.163.5.927, PMID: 16648337

[ref14] Bae G. S. Kim M. S. Jung W. S. Seo S. W. Yun S. W. Kim S. G.. (2010). Inhibition of lipopolysaccharide-induced inflammatory responses by piperine. Eur. J. Pharmacol. 642, 154–162. doi: 10.1016/j.ejphar.2010.05.026, PMID: 20621590

[ref15] Bale J. F. (2009). Fetal infections and brain development. Clin. Perinatol. 36, 639–653. doi: 10.1016/j.clp.2009.06.005, PMID: 19732618

[ref16] Banker S. M. Gu X. Schiller D. Foss-Feig J. H. (2021). Hippocampal contributions to social and cognitive deficits in autism spectrum disorder. Trends Neurosci. 44, 793–807. doi: 10.1016/j.tins.2021.08.005, PMID: 34521563 PMC8484056

[ref17] Bao M. Hofsink N. Plösch T. (2022). LPS vs. poly I:C model: comparison of long-term effects of bacterial and viral maternal immune activation (MIA) on the offspring. Am. J. Physiol. Regul. Integr. Comp. Physiol. 322, R99–R111. doi: 10.1152/AJPREGU.00087.2021, PMID: 34874190 PMC8782664

[ref18] Bargiela S. Steward R. Mandy W. (2016). The experiences of late-diagnosed women with autism spectrum conditions: an investigation of the female autism phenotype. J. Autism Dev. Disord. 46, 3281–3294. doi: 10.1007/s10803-016-2872-8, PMID: 27457364 PMC5040731

[ref19] Barr C. E. Mednick S. A. Munk Jorgensen P. (1990). Exposure to influenza epidemics during gestation and adult schizophrenia: a 40-year study. Arch. Gen. Psychiatry 47, 869–874. doi: 10.1001/archpsyc.1990.01810210077012, PMID: 2393346

[ref20] Bauman M. D. Van de Water J. (2020). Translational opportunities in the prenatal immune environment: promises and limitations of the maternal immune activation model. Neurobiol. Dis. 141:104864:104864. doi: 10.1016/j.nbd.2020.104864, PMID: 32278881 PMC8754471

[ref21] Beery A. K. Zucker I. (2011). Sex bias in neuroscience and biomedical research. Neurosci. Biobehav. Rev. 35, 565–572. doi: 10.1016/j.neubiorev.2010.07.002, PMID: 20620164 PMC3008499

[ref22] Benirschke K. (1990). The placenta in twin gestation. Clin. Obstet. Gynecol. 33, 18–31. doi: 10.1097/00003081-199003000-00006, PMID: 2178836

[ref23] Bergdolt L. Dunaevsky A. (2019). Brain changes in a maternal immune activation model of neurodevelopmental brain disorders. Prog. Neurobiol. 175, 1–19. doi: 10.1016/j.pneurobio.2018.12.002, PMID: 30590095 PMC6413503

[ref24] Berger B. E. Navar-Boggan A. M. Omer S. B. (2011). Congenital rubella syndrome and autism spectrum disorder prevented by rubella vaccination—United States, 2001–2010. BMC Public Health 11, 1–5. doi: 10.1186/1471-2458-11-340, PMID: 21592401 PMC3123590

[ref25] Blomström Å. Karlsson H. Gardner R. Jörgensen L. Magnusson C. Dalman C. (2016). Associations between maternal infection during pregnancy, childhood infections, and the risk of subsequent psychotic disorder - a Swedish cohort study of nearly 2 million individuals. Schizophr. Bull. 42, 125–133. doi: 10.1093/schbul/sbv112, PMID: 26303935 PMC4681563

[ref26] Boksa P. (2008). Maternal infection during pregnancy and schizophrenia. J. Psychiatry Neurosci. 33, 183–185. 18592037 PMC2441883

[ref27] Boksa P. (2010). Effects of prenatal infection on brain development and behavior: a review of findings from animal wmodels. Brain Behav. Immun. 24, 881–897. doi: 10.1016/j.bbi.2010.03.005, PMID: 20230889

[ref28] Bolton J. L. Huff N. C. Smith S. H. Mason S. N. Foster W. M. Auten R. L.. (2013). Maternal stress and effects of prenatal air pollution on offspring mental health outcomes in mice. Environ. Health Perspect. 121, 1075–1082. doi: 10.1289/ehp.1306560, PMID: 23823752 PMC3764088

[ref29] Bordeleau M. Comin C. H. Fernández de Cossío L. Lacabanne C. Freitas-Andrade M. González Ibáñez F.. (2022). Maternal high-fat diet in mice induces cerebrovascular, microglial and long-term behavioural alterations in offspring. Commun. Biol. 5:26. doi: 10.1038/s42003-021-02947-9, PMID: 35017640 PMC8752761

[ref30] Bordeleau M. de Cossío L. F. Lacabanne C. Savage J. C. Vernoux N. Chakravarty M.. (2021). Maternal high-fat diet modifies myelin organization, microglial interactions, and results in social memory and sensorimotor gating deficits in adolescent mouse offspring. Brain Behav. Immun. Health 15:100281:100281. doi: 10.1016/j.bbih.2021.100281, PMID: 34589781 PMC8474164

[ref31] Brašić J. R. Holland J. A. (2007). A qualitative and quantitative review of obstetric complications and autistic disorder. J. Dev. Phys. Disabil. 19, 337–364. doi: 10.1007/s10882-007-9054-8

[ref32] Breach M. R. Lenz K. M. (2022). “Sex differences in neurodevelopmental disorders: a key role for the immune system” in Current topics in behavioral neurosciences eds. Gibson C. Galea L. A. M. (Berlin, Heidelberg: Springer), 165–206. 10.1007/7854_2022_308 PMC10286778 35435643

[ref33] Brehm M. A. Jouvet N. Greiner D. L. Shultz L. D. (2013). Humanized mice for the study of infectious diseases. Curr. Opin. Immunol. 25, 428–435. doi: 10.1016/j.coi.2013.05.012, PMID: 23751490 PMC3775881

[ref34] Brown A. S. (2012). Epidemiologic studies of exposure to prenatal infection and risk of schizophrenia and autism. Dev. Neurobiol. 72, 1272–1276. doi: 10.1002/dneu.22024, PMID: 22488761 PMC3435457

[ref35] Brown A. S. Begg M. D. Gravenstein S. Schaefer C. A. Wyatt R. J. Bresnahan M.. (2004). Serologic evidence of prenatal influenza in the etiology of schizophrenia. Arch. Gen. Psychiatry 61, 774–780. doi: 10.1001/archpsyc.61.8.774, PMID: 15289276

[ref36] Brown A. S. Cohen P. Greenwald S. Susser E. (2000a). Nonaffective psychosis after prenatal exposure to rubella. Am. J. Psychiatr. 157, 438–443. doi: 10.1176/appi.ajp.157.3.438, PMID: 10698821

[ref37] Brown A. S. Cohen P. Harkavy-Friedman J. Babulas V. Malaspina D. Gorman J. M.. (2001). Prenatal rubella, premorbid abnormalities, and adult schizophrenia. Biol. Psychiatry 49, 473–486. doi: 10.1016/S0006-3223(01)01068-X, PMID: 11257233

[ref38] Brown A. S. Schaefer C. A. Quesenberry C. P. Liu L. Babulas V. P. Susser E. S. (2005). Maternal exposure to toxoplasmosis and risk of schizophrenia in adult offspring. Am. J. Psychiatr. 162, 767–773. doi: 10.1176/appi.ajp.162.4.767, PMID: 15800151

[ref39] Brown A. S. Schaefer C. A. Wyatt R. J. Goetz R. Begg M. D. Gorman J. M.. (2000b). Maternal exposure to respiratory infections and adult schizophrenia spectrum disorders: a prospective birth cohort study. Schizophr. Bull. 26, 287–295. doi: 10.1093/oxfordjournals.schbul.a033453, PMID: 10885631

[ref40] Buffington S. A. Di Prisco G. V. Auchtung T. A. Ajami N. J. Petrosino J. F. Costa-Mattioli M. (2016). Microbial reconstitution reverses maternal diet-induced social and synaptic deficits in offspring. Cells 165, 1762–1775. doi: 10.1016/j.cell.2016.06.001, PMID: 27315483 PMC5102250

[ref41] Buka S. L. Tsuang M. T. Torrey E. F. Klebanoff M. A. Wagner R. L. Yolken R. H. (2001). Maternal cytokine levels during pregnancy and adult psychosis. Brain Behav. Immun. 15, 411–420. doi: 10.1006/brbi.2001.0644, PMID: 11782107

[ref42] Campesi I. Marino M. Montella A. Pais S. Franconi F. (2017). Sex differences in estrogen receptor α and β levels and activation status in LPS-stimulated human macrophages. J. Cell. Physiol. 232, 340–345. doi: 10.1002/jcp.25425, PMID: 27171902

[ref43] Carlsson T. Molander F. Taylor M. Jonsson U. Bölte S. (2021). Early environmental risk factors for neurodevelopmental disorders – a systematic review of twin and sibling studies. Dev. Psychopathol. 33, 1448–1495. doi: 10.1017/S0954579420000620, PMID: 32703331 PMC8564717

[ref44] Carter C. J. (2009). Schizophrenia susceptibility genes directly implicated in the life cycles of pathogens: cytomegalovirus, influenza, herpes simplex, rubella, and toxoplasma gondii. Schizophr. Bull. 35, 1163–1182. doi: 10.1093/schbul/sbn054, PMID: 18552348 PMC2762619

[ref45] Chamera K. Trojan E. Kotarska K. Szuster-Głuszczak M. Bryniarska N. Tylek K.. (2021). Role of polyinosinic:polycytidylic acid-induced maternal immune activation and subsequent immune challenge in the behaviour and microglial cell trajectory in adult offspring: a study of the neurodevelopmental model of schizophrenia. Int. J. Mol. Sci. 22:1558. doi: 10.3390/ijms22041558, PMID: 33557113 PMC7913889

[ref46] Chan J. C. Nugent B. M. Bale T. L. (2018). Parental advisory: maternal and paternal stress can impact offspring neurodevelopment. Biol. Psychiatry 83, 886–894. doi: 10.1016/j.biopsych.2017.10.005, PMID: 29198470 PMC5899063

[ref47] Chang Y. C. Cole T. B. Costa L. G. (2018). Prenatal and early-life diesel exhaust exposure causes autism-like behavioral changes in mice. Part. Fibre Toxicol. 15, 1–14. doi: 10.1186/s12989-018-0254-4 29678176 PMC5910592

[ref48] Chang Z. Lichtenstein P. D’Onofrio B. M. Almqvist C. Kuja-Halkola R. Sjölander A.. (2014). Maternal age at childbirth and risk for ADHD in offspring: a population-based cohort study. Int. J. Epidemiol. 43, 1815–1824. doi: 10.1093/ije/dyu204, PMID: 25355726 PMC4276066

[ref49] Cheslack-Postava K. Brown A. S. (2022). Prenatal infection and schizophrenia: a decade of further progress. Schizophr. Res. 247, 7–15. doi: 10.1016/j.schres.2021.05.014, PMID: 34016508 PMC8595430

[ref50] Chess S. Fernandez P. Korn S. (1979). Behavioral consequences of congenital rubella. J. Pediatr. 94, 678–679. doi: 10.1016/S0022-3476(79)80054-2, PMID: 702254

[ref51] Chiarotti F. Venerosi A. (2020). Epidemiology of autism spectrum disorders: a review of worldwide prevalence estimates since 2014. Brain Sci. 10:274. doi: 10.3390/brainsci10050274, PMID: 32370097 PMC7288022

[ref52] Cicero D. C. Martin E. A. Becker T. M. Kerns J. G. (2014). Reinforcement learning deficits in people with schizophrenia persist after extended trials. Psychiatry Res. 220, 760–764. doi: 10.1016/j.psychres.2014.08.013, PMID: 25172610 PMC4258127

[ref53] Clarke M. C. Tanskanen A. Huttunen M. Whittaker J. C. Cannon M. (2009). Evidence for an interaction between familial liability and prenatal exposure to infection in the causation of schizophrenia. Am. J. Psychiatr. 166, 1025–1030. doi: 10.1176/appi.ajp.2009.08010031, PMID: 19487391

[ref54] Cohrs S. (2008). Sleep disturbances in patients with schizophrenia: impact and effect of antipsychotics. CNS Drugs 22, 939–962. doi: 10.2165/00023210-200822110-00004, PMID: 18840034

[ref55] Coiro P. Pollak D. D. (2019). Sex and gender bias in the experimental neurosciences: the case of the maternal immune activation model. Transl. Psychiatry 9, 1–8. doi: 10.1038/s41398-019-0423-8 30765690 PMC6375995

[ref56] Conradt E. Crowell S. E. Cicchetti D. (2021). Using development and psychopathology principles to inform the research domain criteria (RDoC) framework. Dev. Psychopathol. 33, 1521–1525. doi: 10.1017/S0954579421000985, PMID: 35480855 PMC9037759

[ref57] Cotter J. Granger K. Backx R. Hobbs M. Looi C. Y. Barnett J. H. (2018). Social cognitive dysfunction as a clinical marker: a systematic review of meta-analyses across 30 clinical conditions. Neurosci. Biobehav. Rev. 84, 92–99. doi: 10.1016/j.neubiorev.2017.11.014, PMID: 29175518

[ref58] Croen L. A. Qian Y. Ashwood P. Zerbo O. Schendel D. Pinto-Martin J.. (2019). Infection and fever in pregnancy and autism spectrum disorders: findings from the study to explore early development. Autism Res. 12, 1551–1561. doi: 10.1002/aur.2175, PMID: 31317667 PMC7784630

[ref59] Cummings M. J. Baldwin M. R. Abrams D. Jacobson S. D. Meyer B. J. Balough E. M.. (2020). Epidemiology, clinical course, and outcomes of critically ill adults with COVID-19 in new York City: a prospective cohort study. Lancet 395, 1763–1770. doi: 10.1016/S0140-6736(20)31189-2, PMID: 32442528 PMC7237188

[ref60] D’Mello A. (2022). How scientists can counteract their unwitting contributions to autism’s sex bias. Spectrum Autism Research News. Available at: https://www.spectrumnews.org

[ref61] Davis J. Eyre H. Jacka F. N. Dodd S. Dean O. McEwen S.. (2016). A review of vulnerability and risks for schizophrenia: beyond the two-hit hypothesis. Neurosci. Biobehav. Rev. 65, 185–194. doi: 10.1016/j.neubiorev.2016.03.017, PMID: 27073049 PMC4876729

[ref62] Davis J. O. Phelps J. A. Bracha H. S. (1995). Prenatal development of monozygotic twins and concordance for schizophrenia. Schizophr. Bull. 21, 357–366. doi: 10.1093/schbul/21.3.357, PMID: 7481567

[ref63] de Cossío L. F. Guzmán A. Van Der Veldt S. Luheshi G. N. (2017). Prenatal infection leads to ASD-like behavior and altered synaptic pruning in the mouse offspring. Brain Behav. Immun. 63, 88–98. doi: 10.1016/j.bbi.2016.09.028, PMID: 27697456

[ref64] De Lacy N. King B. H. (2013). Revisiting the relationship between autism and schizophrenia: toward an integrated neurobiology. Annu. Rev. Clin. Psychol. 9, 555–587. doi: 10.1146/annurev-clinpsy-050212-185627, PMID: 23537488

[ref65] Desmond M. M. Montgomery J. R. Melnick J. L. Cochran G. G. Verniaud W. (1969). Congenital rubella encephalitis: effects on growth and early development. Am. J. Dis. Child. 118, 30–31. doi: 10.1001/archpedi.1969.02100040032005, PMID: 5815336

[ref66] Deverman B. E. Patterson P. H. (2009). Cytokines and CNS development. Neuron 64, 61–78. doi: 10.1016/j.neuron.2009.09.002, PMID: 19840550

[ref67] Diz-Chaves Y. Astiz M. Bellini M. J. Garcia-Segura L. M. (2013). Prenatal stress increases the expression of proinflammatory cytokines and exacerbates the inflammatory response to LPS in the hippocampal formation of adult male mice. Brain Behav. Immun. 28, 196–206. doi: 10.1016/j.bbi.2012.11.013, PMID: 23207108

[ref68] Diz-Chaves Y. Pernía O. Carrero P. Garcia-Segura L. M. (2012). Prenatal stress causes alterations in the morphology of microglia and the inflammatory response of the hippocampus of adult female mice. J. Neuroinflammation 9, 1–10. doi: 10.1186/1742-2094-9-71 22520439 PMC3409032

[ref69] Dombrowski S. C. Martin R. P. Huttunen M. O. (2003). Association between maternal fever and psychological/behavior outcomes: a hypothesis. Birth defects research (part a): clinical and molecular. Teratology 67, 905–910. doi: 10.1002/bdra.10096 14745927

[ref70] Dreier J. W. Berg-Beckhoff G. Andersen A. M. N. Susser E. Nordentoft M. Strandberg-Larsen K. (2018). Fever and infections during pregnancy and psychosis-like experiences in the offspring at age 11. A prospective study within the Danish National Birth Cohort. Psychol. Med. 48, 426–436. doi: 10.1017/S0033291717001805, PMID: 28735583

[ref71] Edwards M. J. (2007). Hyperthermia in utero due to maternal influenza is an environmental risk factor for schizophrenia. Congenit. Anom. 47, 84–89. doi: 10.1111/j.1741-4520.2007.00151.x, PMID: 17688466

[ref72] Ehsanifar M. Jafari A. J. Nikzad H. Zavareh M. S. Atlasi M. A. Mohammadi H.. (2019). Prenatal exposure to diesel exhaust particles causes anxiety, spatial memory disorders with alters expression of hippocampal pro-inflammatory cytokines and NMDA receptor subunits in adult male mice offspring. Ecotoxicol. Environ. Saf. 176, 34–41. doi: 10.1016/j.ecoenv.2019.03.090, PMID: 30921694

[ref73] Ellman L. M. Yolken R. H. Buka S. L. Torrey E. F. Cannon T. D. (2009). Cognitive functioning prior to the onset of psychosis: the role of fetal exposure to serologically determined influenza infection. Biol. Psychiatry 65, 1040–1047. doi: 10.1016/j.biopsych.2008.12.015, PMID: 19195645 PMC4058882

[ref74] El-Saadi O. Pedersen C. B. McNeil T. F. Saha S. Welham J. O’Callaghan E.. (2004). Paternal and maternal age as risk factors for psychosis: findings from Denmark, Sweden and Australia. Schizophrenia Res. 67, 227–236. doi: 10.1016/S0920-9964(03)00100-2, PMID: 14984882

[ref75] Estes M. L. McAllister A. K. (2016). Maternal immune activation: implications for neuropsychiatric disorders. Science 353, 772–777. doi: 10.1126/science.aag3194, PMID: 27540164 PMC5650490

[ref76] Fan L. W. Pang Y. Lin S. Tien L. T. Ma T. Rhodes P. G.. (2005). Minocycline reduces lipopolysaccharide-induced neurological dysfunction and brain injury in the neonatal rat. J. Neurosci. Res. 82, 71–82. doi: 10.1002/jnr.20623, PMID: 16118791

[ref77] Fang S. Y. Wang S. Huang N. Yeh H. H. Chen C. Y. (2015). Prenatal infection and autism spectrum disorders in childhood: a population-based case-control study in Taiwan. Paediatr. Perinat. Epidemiol. 29, 307–316. doi: 10.1111/ppe.12194, PMID: 25989831

[ref78] Fatemi S. H. Folsom T. D. (2009). The neurodevelopmental hypothesis of schizophrenia, revisited. Schizophr. Bull. 35, 528–548. doi: 10.1093/schbul/sbn187, PMID: 19223657 PMC2669580

[ref79] Flinkkilä E. Keski-Rahkonen A. Marttunen M. Raevuori A. (2016). Prenatal inflammation, infections and mental disorders. Psychopathology 49, 317–333. doi: 10.1159/000448054, PMID: 27529630

[ref80] Foss-Feig J. H. Adkinson B. D. Ji J. L. Yang G. Srihari V. H. McPartland J. C.. (2017). Searching for cross-diagnostic convergence: neural mechanisms governing excitation and inhibition balance in schizophrenia and autism spectrum disorders. Biol. Psychiatry 81, 848–861. doi: 10.1016/j.biopsych.2017.03.005, PMID: 28434615 PMC5436134

[ref81] Fountoulakis K. N. Gonda X. Siamouli M. Panagiotidis P. Moutou K. Nimatoudis I.. (2018). Paternal and maternal age as risk factors for schizophrenia: a case–control study. Int. J. Psychiatry Clin. Pract. 22, 170–176. doi: 10.1080/13651501.2017.1391292, PMID: 29069946

[ref82] Furukawa S. Tsuji N. Sugiyama A. (2019). Morphology and physiology of rat placenta for toxicological evaluation. J. Toxicol. Pathol. 32, 1–17. doi: 10.1293/tox.2018-0042, PMID: 30739991 PMC6361663

[ref83] Ghanizadeh A. (2014). Association of ADHD symptoms severity with higher paternal and lower maternal age of a clinical sample of children. Acta Med. Iran. 52, 49–51. 24658987

[ref84] Giannoni E. Guignard L. Knaup Reymond M. Perreau M. Roth-Kleiner M. Calandra T.. (2011). Estradiol and progesterone strongly inhibit the innate immune response of mononuclear cells in newborns. Infect. Immun. 79, 2690–2698. doi: 10.1128/IAI.00076-11, PMID: 21518785 PMC3191988

[ref85] Gilmore J. H. Jarskog L. F. (1997). Exposure to infection and brain development: cytokines in the pathogenesis of schizophrenia. Schizophr. Res. 24, 365–367. doi: 10.1016/S0920-9964(96)00123-5, PMID: 9134598

[ref86] Goh J. Y. (2020). Evaluation of maternal immune activation with post-weaning social isolation as a potential rat model for schizophrenia. Doctoral dissertation, University of Nottingham (UK) & Monash University (Australia)]. Nottingham.

[ref87] Goines P. E. Croen L. A. Braunschweig D. (2011). Increased mid-gestational IFN-gamma, IL-4, and IL-5 in women giving birth to a child with autism: a case-control study. Molecular. Autism 2:13. doi: 10.1186/2040-2392-2-13, PMID: 21810230 PMC3170586

[ref88] Gold J. M. Waltz J. A. Prentice K. J. Morris S. E. Heerey E. A. (2008). Reward processing in schizophrenia: a deficit in the representation of value. Schizophr. Bull. 34, 835–847. doi: 10.1093/schbul/sbn068, PMID: 18591195 PMC2518641

[ref89] Goldstein J. M. Cherkerzian S. Seidman L. J. Donatelli J.-A. A. L. Remington A. G. Tsuang M. T.. (2014). Prenatal maternal immune disruption and sex-dependent risk for psychoses. Psychol. Med. 44, 3249–3261. doi: 10.1017/S0033291714000683, PMID: 25065485 PMC4477534

[ref90] Greenberg J. A. Bell S. J. Guan Y. Yu Y. H. (2011). Folic acid supplementation and pregnancy: more than just neural tube defect prevention. Rev. Obstet. Gynecol. 4, 52–59. doi: 10.3909/riog0157 22102928 PMC3218540

[ref91] Guisso D. R. Saadeh F. S. Saab D. El Deek J. Chamseddine S. El Hassan H. A.. (2018). Association of autism with maternal infections, perinatal and other risk factors: a case-control study. J. Autism Dev. Disord. 48, 2010–2021. doi: 10.1007/s10803-017-3449-x, PMID: 29332178

[ref92] Guma E. Plitman E. Chakravarty M. M. (2019). The role of maternal immune activation in altering the neurodevelopmental trajectories of offspring: a translational review of neuroimaging studies with implications for autism spectrum disorder and schizophrenia. Neurosci. Biobehav. Rev. 104, 141–157. doi: 10.1016/j.neubiorev.2019.06.020, PMID: 31265870

[ref93] Hameete B. C. Fernández-Calleja J. M. de Groot M. W. Oppewal T. R. Tiemessen M. M. Hogenkamp A.. (2021). The poly (I: C)-induced maternal immune activation model; a systematic review and meta-analysis of cytokine levels in the offspring. Brain Behav. Immun. Health 11:100192. doi: 10.1016/j.bbih.2020.100192, PMID: 34589729 PMC8474626

[ref94] Hamidinejat H. Ghorbanpoor M. Hosseini H. Alavi S. M. Nabavi L. Jalali M. H. R.. (2010). Toxoplasma gondii infection in first-episode and inpatient individuals with schizophrenia. Int. J. Infect. Dis. 14, e978–e981. doi: 10.1016/j.ijid.2010.05.018, PMID: 20843718

[ref95] Han V. X. Patel S. Jones H. F. Dale R. C. (2021). Maternal immune activation and neuroinflammation in human neurodevelopmental disorders. Nat. Rev. Neurol. 17, 564–579. doi: 10.1038/s41582-021-00530-8, PMID: 34341569

[ref96] Hanamsagar R. Alter M. D. Block C. S. Sullivan H. Bolton J. L. Bilbo S. D. (2017). Generation of a microglial developmental index in mice and in humans reveals a sex difference in maturation and immune reactivity. Glia 65, 1504–1520. doi: 10.1002/glia.23176, PMID: 28618077 PMC5540146

[ref97] Harvey L. Boksa P. (2012). Prenatal and postnatal animal models of immune activation: relevance to a range of neurodevelopmental disorders. Dev. Neurobiol. 72, 1335–1348. doi: 10.1002/dneu.22043, PMID: 22730147

[ref98] Harvey L. Boksa P. (2014). Do prenatal immune activation and maternal iron deficiency interact to affect neurodevelopment and early behavior in rat offspring? Brain Behav. Immun. 35, 144–154. doi: 10.1016/j.bbi.2013.09.009, PMID: 24064370

[ref99] Haynes L. (2020). Aging of the immune system: research challenges to enhance the health span of older adults. Front. Aging 1:602108. doi: 10.3389/fragi.2020.602108, PMID: 35822168 PMC9261332

[ref100] Herrero F. Mueller F. S. Gruchot J. Küry P. Weber-Stadlbauer U. Meyer U. (2023). Susceptibility and resilience to maternal immune activation are associated with differential expression of endogenous retroviral elements. Brain Behav. Immun. 107, 201–214. doi: 10.1016/j.bbi.2022.10.006, PMID: 36243285

[ref101] Heyer D. B. Meredith R. M. (2017). Environmental toxicology: sensitive periods of development and neurodevelopmental disorders. Neurotoxicology 58, 23–41. doi: 10.1016/j.neuro.2016.10.017, PMID: 27825840

[ref102] Holingue C. Brucato M. Ladd-Acosta C. Hong X. Volk H. Mueller N. T.. (2020). Interaction between maternal immune activation and antibiotic use during pregnancy and child risk of autism spectrum disorder. Autism Res. 13, 2230–2241. doi: 10.1002/aur.2411, PMID: 33067915 PMC7839062

[ref103] Honein M. A. Dawson A. L. Petersen E. E. Jones A. M. Lee E. H. Yazdy M. M.. (2017). Birth defects among fetuses and infants of US women with evidence of possible Zika virus infection during pregnancy. JAMA 317, 59–68. doi: 10.1001/jama.2016.19006, PMID: 27960197

[ref104] Hooley J. M. (2010). Social factors in schizophrenia. Curr. Dir. Psychol. Sci. 19, 238–242. doi: 10.1177/0963721410377597, PMID: 36921404

[ref105] Hornig M. Bresnahan M. A. Che X. Schultz A. F. Ukaigwe J. E. Eddy M. L.. (2018). Prenatal fever and autism risk. Mol. Psychiatry 23, 759–766. doi: 10.1038/mp.2017.119, PMID: 28607458 PMC5822459

[ref106] Hsiao E. Y. Patterson P. H. (2011). Activation of the maternal immune system induces endocrine changes in the placenta via IL-6. Brain Behav. Immun. 25, 604–615. doi: 10.1016/j.bbi.2010.12.017, PMID: 21195166 PMC3081363

[ref107] Hsiao E. Y. Patterson P. H. (2012). Placental regulation of maternal-fetal interactions and brain development. Dev. Neurobiol. 72, 1317–1326. doi: 10.1002/dneu.22045, PMID: 22753006

[ref108] Hui C. W. Vecchiarelli H. A. Gervais É. Luo X. Michaud F. Scheefhals L.. (2020). Sex differences of microglia and synapses in the hippocampal dentate gyrus of adult mouse offspring exposed to maternal immune activation. Front. Cell. Neurosci. 14:558181. doi: 10.3389/fncel.2020.558181, PMID: 33192308 PMC7593822

[ref109] Hutton J. (2016). Does rubella cause autism: a 2015 reappraisal? Front. Hum. Neurosci. 10:25. doi: 10.3389/fnhum.2016.00025, PMID: 26869906 PMC4734211

[ref110] Hvolby A. (2014). Associations of sleep disturbance with ADHD: implications for treatment. ADHD 7, 1–18. doi: 10.1007/S12402-014-0151-0, PMID: 25127644 PMC4340974

[ref111] Hvolgaard Mikkelsen S. Olsen J. Bech B. H. Obel C. (2017). Parental age and attention-deficit/hyperactivity disorder (ADHD). Int. J. Epidemiol. 46, dyw073–dyw420. doi: 10.1093/ije/dyw073, PMID: 27170763

[ref112] Jiang H. Y. Xu L. L. Shao L. Xia R. M. Yu Z. H. Ling Z. X.. (2016). Maternal infection during pregnancy and risk of autism spectrum disorders: a systematic review and meta-analysis. Brain Behav. Immun. 58, 165–172. doi: 10.1016/j.bbi.2016.06.005, PMID: 27287966

[ref113] Jones P. B. Rantakallio P. Hartikainen A. L. Isohanni M. Sipila P. (1998). Schizophrenia as a long-term outcome of pregnancy, delivery, and perinatal complications: a 28-year follow-up of the 1966 North Finland general population birth cohort. Am. J. Psychiatr. 155, 355–364. doi: 10.1176/ajp.155.3.355, PMID: 9501745

[ref114] Jónsson H. Sulem P. Kehr B. Kristmundsdottir S. Zink F. Hjartarson E.. (2017). Parental influence on human germline de novo mutations in 1,548 trios from Iceland. Nature 549, 519–522. doi: 10.1038/nature24018, PMID: 28959963

[ref115] Kalish B. T. Kim E. Finander B. Duffy E. E. Kim H. Gilman C. K.. (2021). Maternal immune activation in mice disrupts proteostasis in the fetal brain. Nat. Neurosci. 24, 204–213. doi: 10.1038/s41593-020-00762-9, PMID: 33361822 PMC7854524

[ref116] Kaskie R. E. Graziano B. Ferrarelli F. (2017). Schizophrenia and sleep disorders: Links, risks, and management challenges. Nat. Sci. Sleep 9, 227–239. doi: 10.2147/NSS.S121076, PMID: 29033618 PMC5614792

[ref117] Kentner A. C. Bilbo S. D. Brown A. S. Hsiao E. Y. McAllister A. K. Meyer U.. (2019). Maternal immune activation: reporting guidelines to improve the rigor, reproducibility, and transparency of the model. Neuropsychopharmacology 44, 245–258. doi: 10.1038/s41386-018-0185-7, PMID: 30188509 PMC6300528

[ref118] Kern J. K. Geier D. A. King P. G. Sykes L. K. Mehta J. A. Geier M. R. (2015). Shared brain connectivity issues, symptoms, and comorbidities in autism spectrum disorder, attention deficit/hyperactivity disorder, and tourette syndrome. Brain Connect. 5, 321–335. doi: 10.1089/brain.2014.0324, PMID: 25602622

[ref119] Khandaker G. M. Zimbron J. Lewis G. Jones P. B. (2013). Prenatal maternal infection, neurodevelopment and adult schizophrenia: a systematic review of population-based studies. Psychol. Med. 43, 239–257. doi: 10.1017/S0033291712000736, PMID: 22717193 PMC3479084

[ref120] Kim S. Kim H. Yim Y. S. Ha S. Atarashi K. Tan T. G.. (2017). Maternal gut bacteria promote neurodevelopmental abnormalities in mouse offspring. Nature 549, 528–532. doi: 10.1038/nature23910, PMID: 28902840 PMC5870873

[ref121] Klein S. L. Flanagan K. L. (2016). Sex differences in immune responses. Nat. Rev. Immunol. 16, 626–638. doi: 10.1038/nri.2016.90, PMID: 27546235

[ref122] Klein S. L. Schwarz J. M. (2018). Sex-specific regulation of peripheral and central immune responses. Oxford Research Encyclopedia of Neuroscience. 1–32. doi: 10.1093/acrefore/9780190264086.013.223

[ref123] Knuesel I. Chicha L. Britschgi M. Schobel S. A. Bodmer M. Hellings J. A.. (2014). Maternal immune activation and abnormal brain development across CNS disorders. Nat. Rev. Neurol. 10, 643–660. doi: 10.1038/nrneurol.2014.187, PMID: 25311587

[ref124] Kong A. Frigge M. L. Masson G. Besenbacher S. Sulem P. Magnusson G.. (2012). Rate of de novo mutations and the importance of father’s age to disease risk. Nature 488, 471–475. doi: 10.1038/nature11396, PMID: 22914163 PMC3548427

[ref125] Konofal E. Lecendreux M. Cortese S. (2010). Sleep and ADHD. Sleep Med. 11, 652–658. doi: 10.1016/j.sleep.2010.02.012, PMID: 20620109

[ref126] Kovats S. (2015). Estrogen receptors regulate innate immune cells and signaling pathways. Cell. Immunol. 294, 63–69. doi: 10.1016/j.cellimm.2015.01.018, PMID: 25682174 PMC4380804

[ref127] Krakowiak P. Walker C. K. Bremer A. A. Baker A. S. Ozonoff S. Hansen R. L.. (2012). Maternal metabolic conditions and risk for autism and other neurodevelopmental disorders. Pediatrics 129, e1121–e1128. doi: 10.1542/peds.2011-2583, PMID: 22492772 PMC3340592

[ref128] Labrousse V. F. Leyrolle Q. Amadieu C. Aubert A. Séré A. Coutureau E.. (2018). Dietary omega-3 deficiency exacerbates inflammation and reveals spatial memory deficits in mice exposed to lipopolysaccharide during gestation. Brain Behav. Immun. 73, 427–440. doi: 10.1016/j.bbi.2018.06.004, PMID: 29879442

[ref129] Lai M. C. Lombardo M. V. Ruigrok A. N. Chakrabarti B. Auyeung B. Szatmari P.. (2017). Quantifying and exploring camouflaging in men and women with autism. Autism 21, 690–702. doi: 10.1177/1362361316671012, PMID: 27899710 PMC5536256

[ref130] Lazic S. E. Essioux L. (2013). Improving basic and translational science by accounting for litter-to-litter variation in animal models. BMC Neurosci. 14, 1–11. doi: 10.1186/1471-2202-14-37, PMID: 23522086 PMC3661356

[ref131] Lee B. K. Magnusson C. Gardner R. M. Blomström Å. Newschaffer C. J. Burstyn I.. (2015). Maternal hospitalization with infection during pregnancy and risk of autism spectrum disorders. Brain Behav. Immun. 44, 100–105. doi: 10.1016/j.bbi.2014.09.001, PMID: 25218900 PMC4418173

[ref132] Li D. Law S. Andermann L. (2012). Association between degrees of social defeat and themes of delusion in patients with schizophrenia from immigrant and ethnic minority backgrounds. Transcult. Psychiatry 49, 735–749. doi: 10.1177/1363461512464625, PMID: 23138195

[ref133] Liew Z. Ritz B. Rebordosa C. Lee P. C. Olsen J. (2014). Acetaminophen use during pregnancy, behavioral problems, and hyperkinetic disorders. JAMA Pediatr. 168, 313–320. doi: 10.1001/jamapediatrics.2013.4914, PMID: 24566677

[ref134] Liew Z. Ritz B. Virk J. Olsen J. (2016). Maternal use of acetaminophen during pregnancy and risk of autism spectrum disorders in childhood: a Danish national birth cohort study. Autism Res. 9, 951–958. doi: 10.1002/aur.1591, PMID: 26688372

[ref135] Lim K. O. Beal D. M. Harvey R. L. Myers T. Lane B. Sullivan E. V.. (1995). Brain dysmorphology in adults with congenital rubella plus schizophrenialike symptoms. Biol. Psychiatry 37, 764–776. doi: 10.1016/0006-3223(94)00219-S, PMID: 7647161

[ref136] Limosin F. Rouillon F. Payan C. Cohen J. M. Strub N. (2003). Prenatal exposure to influenza as a risk factor for adult schizophrenia. Acta Psychiatr. Scand. 107, 331–335. doi: 10.1034/j.1600-0447.2003.00052.x, PMID: 12752028

[ref137] Lindquist J. M. Plotkin S. A. Shaw L. Gilden R. V. Williams M. L. (1965). Congenital rubella syndrome as a systemic infection. Studies of affected infants born in Philadelphia, U.S.A. Br. Med. J. 2, 1401–1406. doi: 10.1136/bmj.2.5475.1401, PMID: 5892115 PMC1847241

[ref138] Lintas C. Altieri L. Lombardi F. Sacco R. Persico A. M. (2010). Association of autism with polyomavirus infection in postmortem brains. J. Neurovirol. 16, 141–149. doi: 10.3109/13550281003685839, PMID: 20345322

[ref139] Liu T. Zhang L. Joo D. Sun S. C. (2017). NF-κB signaling in inflammation. Signal Transduct. Target. Ther. 2:17023. doi: 10.1038/sigtrans.2017.23, PMID: 29158945 PMC5661633

[ref140] Londero A. P. Rossetti E. Pittini C. Cagnacci A. Driul L. (2019). Maternal age and the risk of adverse pregnancy outcomes: a retrospective cohort study. BMC Pregnancy Childbirth 19:261. doi: 10.1186/s12884-019-2400-x, PMID: 31337350 PMC6651936

[ref141] Lopez-Castroman J. Gómez D. D. Belloso J. J. C. Fernandez-Navarro P. Perez-Rodriguez M. M. Villamor I. B.. (2010). Differences in maternal and paternal age between schizophrenia and other psychiatric disorders. Schizophr. Res. 116, 184–190. doi: 10.1016/j.schres.2009.11.006, PMID: 19945257

[ref142] López-Rivera J. A. Pérez-Palma E. Symonds J. Lindy A. S. McKnight D. A. Leu C.. (2020). A catalogue of new incidence estimates of monogenic neurodevelopmental disorders caused by de novo variants. Brain 143, 1099–1105. doi: 10.1093/brain/awaa051, PMID: 32168371 PMC7174049

[ref143] Magnus P. Irgens L. M. Haug K. Nystad W. Skjaerven R. Stoltenberg C.. (2006). Cohort profile: the Norwegian mother and child cohort study (MoBa). Int. J. Epidemiol. 35, 1146–1150. doi: 10.1093/ije/dyl170, PMID: 16926217

[ref144] Mahic M. Che X. Susser E. Levin B. Reichborn-Kjennerud T. Magnus P.. (2017). Epidemiological and serological investigation into the role of gestational maternal influenza virus infection and autism spectrum disorders. MSphere 2:e00159-17. doi: 10.1128/mSphere.00159-17, PMID: 28656175 PMC5480032

[ref145] Makris G. Eleftheriades A. Pervanidou P. (2022). Early life stress, hormones, and neurodevelopmental disorders. Horm. Res. Paediatr. 96, 17–24. doi: 10.1159/000523942 35259742

[ref146] Martin S. M. Malkinson T. J. Veale W. L. Pittman Q. J. (1995). Fever in pregnant, parturient, and lactating rats. Am. J. Phys. Regul. Integr. Comp. Phys. 268, R919–R923. doi: 10.1152/ajpregu.1995.268.4.R919, PMID: 7733402

[ref147] Massarali A. Adhya D. Srivastava D. P. Baron-Cohen S. Kotter M. R. (2022). Virus-induced maternal immune activation as an environmental factor in the etiology of autism and schizophrenia. Front. Neurosci. 16:834058. doi: 10.3389/fnins.2022.834058, PMID: 35495047 PMC9039720

[ref148] Matcovitch-Natan O. Winter D. R. Giladi A. Vargas Aguilar S. Spinrad A. Sarrazin S.. (2016). Microglia development follows a stepwise program to regulate brain homeostasis. Science 353:aad8670. doi: 10.1126/science.aad8670, PMID: 27338705

[ref149] Mawson A. R. Croft A. M. (2019). Rubella virus infection, the congenital rubella syndrome, and the link to autism. Int. J. Environ. Res. Public Health 16:3543. doi: 10.3390/ijerph16193543, PMID: 31546693 PMC6801530

[ref150] McCarroll S. A. Hyman S. E. (2013). Progress in the genetics of polygenic brain disorders: significant new challenges for neurobiology. Neuron 80, 578–587. doi: 10.1016/j.neuron.2013.10.046, PMID: 24183011 PMC4066986

[ref151] Mednick S. Huttunen M. O. Machón R. A. (1994). Prenatal influenza infections and adult schizophrenia. Schizophr. Bull. 20, 263–267. doi: 10.1093/schbul/20.2.263, PMID: 8085130

[ref152] Mednick S. A. Machon R. A. Huttunen M. O. Bonett D. (1988). Adult schizophrenia following prenatal exposure to an influenza epidemic. Arch. Gen. Psychiatry 45, 189–192. doi: 10.1001/archpsyc.1988.01800260109013, PMID: 3337616

[ref153] Meyer U. (2019). Neurodevelopmental resilience and susceptibility to maternal immune activation. Trends Neurosci. 42, 793–806. doi: 10.1016/j.tins.2019.08.001, PMID: 31493924

[ref154] Meyer U. Feldon J. Fatemi S. H. (2009). In-vivo rodent models for the experimental investigation of prenatal immune activation effects in neurodevelopmental brain disorders. Neurosci. Biobehav. Rev. 33, 1061–1079. doi: 10.1016/j.neubiorev.2009.05.001, PMID: 19442688

[ref155] Miller G. E. Borders A. E. Crockett A. H. Ross K. M. Qadir S. Keenan-Devlin L.. (2017). Maternal socioeconomic disadvantage is associated with transcriptional indications of greater immune activation and slower tissue maturation in placental biopsies and newborn cord blood. Brain Behav. Immun. 64, 276–284. doi: 10.1016/j.bbi.2017.04.014, PMID: 28434870 PMC5493326

[ref156] Minakova E. Warner B. B. (2018). Maternal immune activation, central nervous system development and behavioral phenotypes. Birth Defects Res. 110, 1539–1550. doi: 10.1002/bdr2.1416, PMID: 30430765

[ref157] Money K. M. Barke T. L. Serezani A. Gannon M. Garbett K. A. Aronoff D. M.. (2018). Gestational diabetes exacerbates maternal immune activation effects in the developing brain. Mol. Psychiatry 23, 1920–1928. doi: 10.1038/mp.2017.191, PMID: 28948973 PMC6459194

[ref158] Moreno J. L. Kurita M. Holloway T. López J. Cadagan R. Martínez-Sobrido L.. (2011). Maternal influenza viral infection causes schizophrenia-like alterations of 5-HT2A and mGlu2 receptors in the adult offspring. J. Neurosci. 31, 1863–1872. doi: 10.1523/JNEUROSCI.4230-10.2011, PMID: 21289196 PMC3037097

[ref159] Morris-Rosendahl D. J. Crocq M. A. (2020). Neurodevelopmental disorders-the history and future of a diagnostic concept. Dialogues Clin. Neurosci. 22, 65–72. doi: 10.31887/DCNS.2020.22.1/macrocq, PMID: 32699506 PMC7365295

[ref160] Mortensen P. B. Nørgaard-Pedersen B. Waltoft B. L. Sørensen T. L. Hougaard D. Torrey E. F.. (2007). Toxoplasma gondii as a risk factor for early-onset schizophrenia: analysis of filter paper blood samples obtained at birth. Biol. Psychiatry 61, 688–693. doi: 10.1016/j.biopsych.2006.05.024, PMID: 16920078

[ref162] Myles H. Myles N. Antic N. A. Adams R. Chandratilleke M. Liu D.. (2016). Obstructive sleep apnea and schizophrenia: a systematic review to inform clinical practice. Schizophr. Res. 170, 222–225. doi: 10.1016/j.schres.2015.11.014, PMID: 26621003

[ref163] Nair A. Jolliffe M. Lograsso Y. S. S. Bearden C. E. (2020). A review of default mode network connectivity and its association with social cognition in adolescents with autism spectrum disorder and early-onset psychosis. Front. Psych. 11:614. doi: 10.3389/FPSYT.2020.00614/BIBTEX, PMID: 32670121 PMC7330632

[ref164] Nielsen P. R. Laursen T. M. Mortensen P. B. (2013). Association between parental hospital-treated infection and the risk of schizophrenia in adolescence and early adulthood. Schizophr. Bull. 39, 230–237. doi: 10.1093/schbul/sbr149, PMID: 22021661 PMC3523915

[ref165] Nielsen P. R. Meyer U. Mortensen P. B. (2016). Individual and combined effects of maternal anemia and prenatal infection on risk for schizophrenia in offspring. Schizophr. Res. 172, 35–40. doi: 10.1016/j.schres.2016.02.025, PMID: 26899344

[ref166] Nijmeijer J. S. Minderaa R. B. Buitelaar J. K. Mulligan A. Hartman C. A. Hoekstra P. J. (2008). Attention-deficit/hyperactivity disorder and social dysfunctioning. Clin. Psychol. Rev. 28, 692–708. doi: 10.1016/j.cpr.2007.10.003, PMID: 18036711

[ref167] Ning H. Wang H. Zhao L. Zhang C. Li X. Y. Chen Y. H.. (2008). Maternally-administered lipopolysaccharide (LPS) increases tumor necrosis factor alpha in fetal liver and fetal brain: its suppression by low-dose LPS pretreatment. Toxicol. Lett. 176, 13–19. doi: 10.1016/j.toxlet.2007.08.002, PMID: 18060704

[ref169] Ornoy A. Weinstein-Fudim L. Ergaz Z. (2015). Prenatal factors associated with autism spectrum disorder (ASD). Reprod. Toxicol. 56, 155–169. doi: 10.1016/j.reprotox.2015.05.007, PMID: 26021712

[ref170] Osborne B. F. Turano A. Schwarz J. M. (2018). Sex differences in the neuroimmune system. Curr. Opin. Behav. Sci. 23, 118–123. doi: 10.1016/j.cobeha.2018.05.007, PMID: 30014014 PMC6044467

[ref171] Oyarzábal A. Musokhranova U. Barros L. García Cazorla A. (2021). Energy metabolism in childhood neurodevelopmental disorders. EBioMedicine 69:103474. doi: 10.1016/j.ebiom.2021.103474, PMID: 34256347 PMC8324816

[ref172] Paolicelli R. C. Bolasco G. Pagani F. Maggi L. Scianni M. Panzanelli P.. (2011). Synaptic pruning by microglia is necessary for normal brain development. Science 333, 1456–1458. doi: 10.1126/science.1202529, PMID: 21778362

[ref173] Parker C. C. Chen H. Flagel S. B. Geurts A. M. Richards J. B. Robinson T. E.. (2014). Rats are the smart choice: rationale for a renewed focus on rats in behavioral genetics. Neuropharmacology 76, 250–258. doi: 10.1016/j.neuropharm.2013.05.047, PMID: 23791960 PMC3823679

[ref174] Patel S. Masi A. Dale R. C. Whitehouse A. J. O. Pokorski I. Alvares G. A.. (2018). Social impairments in autism spectrum disorder are related to maternal immune history profile. Mol. Psychiatry 23, 1794–1797. doi: 10.1038/mp.2017.201, PMID: 28993711

[ref175] Patrono E. Svoboda J. Stuchlík A. (2021). Schizophrenia, the gut microbiota, and new opportunities from optogenetic manipulations of the gut-brain axis. Behav. Brain Funct. 17, 1–21. doi: 10.1186/s12993-021-00180-2 34158061 PMC8218443

[ref176] Pearce B. D. (2001). Schizophrenia and viral infection during neurodevelopment: a focus on mechanisms. Mol. Psychiatry 6, 634–646. doi: 10.1038/sj.mp.4000956, PMID: 11673791

[ref177] Penna E. Pizzella A. Cimmino F. Trinchese G. Cavaliere G. Catapano A.. (2020). Neurodevelopmental disorders: effect of high-fat diet on synaptic plasticity and mitochondrial functions. Brain Sci. 10:805. doi: 10.3390/brainsci10110805, PMID: 33142719 PMC7694125

[ref178] Perepa P. (2014). Cultural basis of social “deficits” in autism spectrum disorders. Eur. J. Spec. Needs Educ. 29, 313–326. doi: 10.1080/08856257.2014.908024, PMID: 34167580

[ref179] Pinares-Garcia P. Stratikopoulos M. Zagato A. Loke H. Lee J. (2018). Sex: a significant risk factor for neurodevelopmental and neurodegenerative disorders. Brain Sci. 8:154. doi: 10.3390/brainsci8080154, PMID: 30104506 PMC6120011

[ref180] Polyak A. Rosenfeld J. A. Girirajan S. (2015). An assessment of sex bias in neurodevelopmental disorders. Genome Med. 7, 94–11. doi: 10.1186/s13073-015-0216-5, PMID: 26307204 PMC4549901

[ref181] Pomar L. Malinger G. Benoist G. Carles G. Ville Y. Rousset D.. (2017). Association between Zika virus and fetopathy: a prospective cohort study in French Guiana. Ultrasound Obstet. Gynecol. 49, 729–736. doi: 10.1002/uog.17404, PMID: 28078779

[ref182] Pope J. W. Kern R. S. (2006). An “errorful” learning deficit in schizophrenia? J. Clin. Exp. Neuropsychol. 28, 101–110. doi: 10.1080/13803390490918138, PMID: 16448979

[ref183] Porcelli S. Van Der Wee N. van der Werff S. Aghajani M. Glennon J. C. van Heukelum S.. (2019). Social brain, social dysfunction and social withdrawal. Neurosci. Biobehav. Rev. 97, 10–33. doi: 10.1016/j.neubiorev.2018.09.012, PMID: 30244163

[ref184] Rapoport J. L. Giedd J. N. Gogtay N. (2012). Neurodevelopmental model of schizophrenia: update 2012. Mol. Psychiatry 17, 1228–1238. doi: 10.1038/mp.2012.23, PMID: 22488257 PMC3504171

[ref185] Reisinger S. Khan D. Kong E. Berger A. Pollak A. Pollak D. D. (2015). The poly(I:C)-induced maternal immune activation model in preclinical neuropsychiatric drug discovery. Pharmacol. Ther. 149, 213–226. doi: 10.1016/j.pharmthera.2015.01.001, PMID: 25562580

[ref186] Richard C. Lewis E. D. Goruk S. Wadge E. Curtis J. M. Jacobs R. L.. (2017). Feeding a mixture of choline forms to lactating dams improves the development of the immune system in Sprague-Dawley rat offspring. Nutrients 9:567. doi: 10.3390/nu9060567, PMID: 28574475 PMC5490546

[ref187] Richardson A. J. Ross M. A. (2000). Fatty acid metabolism in neurodevelopmental disorder: a new perspective on associations between attention-deficit/hyperactivity disorder, dyslexia, dyspraxia and the autistic spectrum. Prostaglandins Leukot. Essent. Fatty Acids 63, 1–9. doi: 10.1054/plef.2000.0184, PMID: 10970706

[ref188] Robbins J. R. Bakardjiev A. I. (2012). Pathogens and the placental fortress. Curr. Opin. Microbiol. 15, 36–43. doi: 10.1016/j.mib.2011.11.006, PMID: 22169833 PMC3265690

[ref189] Robinson D. P. Klein S. L. (2012). Pregnancy and pregnancy-associated hormones alter immune responses and disease pathogenesis. Horm. Behav. 62, 263–271. doi: 10.1016/j.yhbeh.2012.02.023, PMID: 22406114 PMC3376705

[ref190] Ronald A. Pennell C. E. Whitehouse A. J. (2011). Prenatal maternal stress associated with ADHD and autistic traits in early childhood. Front. Psychol. 1:223. doi: 10.3389/fpsyg.2010.00223 21833278 PMC3153828

[ref191] Ryan B. C. Vandenbergh J. G. (2002). Intrauterine position effects. Neurosci. Biobehav. Rev. 26, 665–678. doi: 10.1016/S0149-7634(02)00038-6, PMID: 12479841

[ref192] Sandin S. Hultman C. M. Kolevzon A. Gross R. MacCabe J. H. Reichenberg A. (2012). Advancing maternal age is associated with increasing risk for autism: a review and meta-analysis. J. Am. Acad. Child Adolesc. Psychiatry 51, 477–486.e1. doi: 10.1016/j.jaac.2012.02.018, PMID: 22525954

[ref193] Saper C. B. Stornetta R. L. (2015). “Central autonomic system” in The rat nervous system ed. George P. (Cambridge, MA: Academic Press), 629–673.

[ref194] Sarkar S. Heise M. T. (2019). Mouse models as resources for studying infectious diseases. Clin. Ther. 41, 1912–1922. doi: 10.1016/j.clinthera.2019.08.010, PMID: 31540729 PMC7112552

[ref195] Savla G. N. Vella L. Armstrong C. C. Penn D. L. Twamley E. W. (2013). Deficits in domains of social cognition in schizophrenia: a meta-analysis of the empirical evidence. Schizophr. Bull. 39, 979–992. doi: 10.1093/schbul/sbs080, PMID: 22949733 PMC3756768

[ref196] Schafer D. P. Lehrman E. K. Kautzman A. G. Koyama R. Mardinly A. R. Yamasaki R.. (2012). Microglia sculpt postnatal neural circuits in an activity and complement-dependent manner. Neuron 74, 691–705. doi: 10.1016/j.neuron.2012.03.026, PMID: 22632727 PMC3528177

[ref197] Schwarz J. M. Bilbo S. D. (2011a). LPS elicits a much larger and broader inflammatory response than Escherichia coli infection within the hippocampus of neonatal rats. Neurosci. Lett. 497, 110–115. doi: 10.1016/j.neulet.2011.04.042, PMID: 21536105 PMC3103622

[ref198] Schwarz J. M. Bilbo S. D. (2011b). “The immune system and the developing brain” in Colloquium series on the developing brain (San Rafael, CA: Morgan & Claypool Life Sciences), 1–128.

[ref199] Schwarz J. M. Sholar P. W. Bilbo S. D. (2012). Sex differences in microglial colonization of the developing rat brain. J. Neurochem. 120, 948–963. doi: 10.1111/j.1471-4159.2011.07630.x, PMID: 22182318 PMC3296888

[ref200] Sciberras E. Mulraney M. Silva D. Coghill D. (2017). Prenatal risk factors and the etiology of ADHD—review of existing evidence. Curr. Psychiatry Rep. 19, 1–8. doi: 10.1007/s11920-017-0753-2, PMID: 28091799

[ref201] Sham P. C. O’Callaghan E. Takei N. Murray G. K. Hare E. H. Murray R. M. (1992). Schizophrenia following prenatal exposure to influenza epidemics between 1939 and 1960. Br. J. Psychiatry 160, 461–466. doi: 10.1192/bjp.160.4.461, PMID: 1294066

[ref202] Shansky R. M. Murphy A. Z. (2021). Considering sex as a biological variable will require a global shift in science culture. Nat. Neurosci. 24, 457–464. doi: 10.1038/s41593-021-00806-8, PMID: 33649507 PMC12900283

[ref203] Shelton A. R. Malow B. (2021). Neurodevelopmental disorders commonly presenting with sleep disturbances. Neurotherapeutics 18, 156–169. doi: 10.1007/s13311-020-00982-8, PMID: 33403472 PMC8116361

[ref204] Shepherd R. Cheung A. S. Pang K. Saffery R. Novakovic B. (2021). Sexual dimorphism in innate immunity: the role of sex hormones and epigenetics. Front. Immunol. 11:604000. doi: 10.3389/fimmu.2020.604000, PMID: 33584674 PMC7873844

[ref205] Sherer M. L. Posillico C. K. Schwarz J. M. (2017). An examination of changes in maternal neuroimmune function during pregnancy and the postpartum period. Brain Behav. Immun. 66, 201–209. doi: 10.1016/j.bbi.2017.06.016, PMID: 28669797 PMC6348474

[ref206] Shi L. Fatemi S. H. Sidwell R. W. Patterson P. H. (2003). Maternal influenza infection causes marked behavioral and pharmacological changes in the offspring. J. Neurosci. 23, 297–302. doi: 10.1523/JNEUROSCI.23-01-00297.2003, PMID: 12514227 PMC6742135

[ref207] Shi L. Tu N. Patterson P. H. (2005). Maternal influenza infection is likely to alter fetal brain development indirectly: the virus is not detected in the fetus. Int. J. Dev. Neurosci. 23, 299–305. doi: 10.1016/j.ijdevneu.2004.05.005, PMID: 15749254

[ref208] Shuid A. N. Jayusman P. A. Shuid N. Ismail J. Nor N. K. Mohamed I. N. (2021). Association between viral infections and risk of autistic disorder: an overview. Int. J. Environ. Res. Public Health 18, 1–17. doi: 10.3390/ijerph18062817, PMID: 33802042 PMC7999368

[ref209] Singh K. Singh I. N. Diggins E. Connors S. L. Karim M. A. Lee D.. (2020). Developmental regression and mitochondrial function in children with autism. Ann. Clin. Transl. Neurol. 7, 683–694. doi: 10.1002/acn3.51034, PMID: 32343046 PMC7261756

[ref210] Smolders S. Notter T. Smolders S. M. Rigo J. M. Brône B. (2018). Controversies and prospects about microglia in maternal immune activation models for neurodevelopmental disorders. Brain Behav. Immun. 73, 51–65. doi: 10.1016/j.bbi.2018.06.001, PMID: 29870753

[ref211] Solek C. M. Farooqi N. Verly M. Lim T. K. Ruthazer E. S. (2018). Maternal immune activation in neurodevelopmental disorders. Dev. Dyn. 247, 588–619. doi: 10.1002/dvdy.24612, PMID: 29226543

[ref212] Song J. W. Chung K. C. (2010). Observational studies: cohort and case-control studies. Plast. Reconstr. Surg. 126, 2234–2242. doi: 10.1097/PRS.0b013e3181f44abc, PMID: 20697313 PMC2998589

[ref213] Sørensen H. J. Mortensen E. L. Reinisch J. M. Mednick S. A. (2009). Association between prenatal exposure to bacterial infection and risk of schizophrenia. Schizophr. Bull. 35, 631–637. doi: 10.1093/schbul/sbn121, PMID: 18832344 PMC2669577

[ref214] Spini V. B. Ferreira F. R. Gomes A. O. Duarte R. M. F. Oliveira V. H. S. Costa N. B.. (2021). Maternal immune activation with H1N1 or toxoplasma gondii antigens induces behavioral impairments associated with mood disorders in rodents. Neuropsychobiology 80, 234–241. doi: 10.1159/000510791, PMID: 33070134

[ref215] Staikova E. Gomes H. Tartter V. McCabe A. Halperin J. M. (2013). Pragmatic deficits and social impairment in children with ADHD. J. Child Psychol. Psychiatry Allied Discip. 54, 1275–1283. doi: 10.1111/jcpp.12082, PMID: 23682627 PMC3648855

[ref216] Stein M. A. Weiss M. Hlavaty L. (2012). ADHD treatments, sleep, and sleep problems: complex associations. Neurotherapeutics 9, 509–517. doi: 10.1007/s13311-012-0130-0, PMID: 22718078 PMC3441938

[ref217] Stoltenberg C. Schjølberg S. Bresnahan M. Hornig M. Hirtz D. Dahl C.. (2010). The autism birth cohort: a paradigm for gene-environment-timing research. Mol. Psychiatry 15, 676–680. doi: 10.1038/mp.2009.143, PMID: 20571529 PMC2892398

[ref218] Sullivan E. L. Riper K. M. Lockard R. Valleau J. C. (2015). Maternal high-fat diet programming of the neuroendocrine system and behavior. Horm. Behav. 76, 153–161. doi: 10.1016/j.yhbeh.2015.04.008, PMID: 25913366 PMC4619177

[ref219] Supekar K. Uddin L. Q. Khouzam A. Phillips J. Gaillard W. D. Kenworthy L. E.. (2013). Brain hyperconnectivity in children with autism and its links to social deficits. Cell Rep. 5, 738–747. doi: 10.1016/j.celrep.2013.10.001, PMID: 24210821 PMC3894787

[ref220] Swisher C. N. Swisher L. (1975). Congenital rubella and autistic behavior. N. Engl. J. Med. 293:198. 1134536

[ref221] Takei N. Mortensen P. B. Klæning U. Murray R. M. Sham P. C. O’Callaghan E.. (1996). Relationship between in utero exposure to influenza epidemics and risk of schizophrenia in Denmark. Biol. Psychiatry 40, 817–824. doi: 10.1016/0006-3223(95)00592-7, PMID: 8896767

[ref222] Takeyama J. Suzuki T. Inoue S. Kaneko C. Nagura H. Harada N.. (2001). Expression and cellular localization of estrogen receptors alpha and beta in the human fetus. J. Clin. Endocrinol. Metab. 86, 2258–2262. doi: 10.1210/jcem.86.5.7447, PMID: 11344236

[ref223] Tanabe S. Yamashita T. (2018). The role of immune cells in brain development and neurodevelopmental diseases. Int. Immunol. 30, 437–444. doi: 10.1093/intimm/dxy041, PMID: 29917120

[ref224] Tay T. L. Savage J. C. Hui C. W. Bisht K. Tremblay M. È. (2017). Microglia across the lifespan: from origin to function in brain development, plasticity and cognition. J. Physiol. 595, 1929–1945. doi: 10.1113/JP272134, PMID: 27104646 PMC5350449

[ref225] Tick B. Bolton P. Happé F. Rutter M. Rijsdijk F. (2016). Heritability of autism spectrum disorders: a meta-analysis of twin studies. J. Child Psychol. Psychiatry 57, 585–595. doi: 10.1111/jcpp.12499, PMID: 26709141 PMC4996332

[ref226] Tioleco N. Silberman A. E. Stratigos K. Banerjee-Basu S. Spann M. N. Whitaker A. H.. (2021). Prenatal maternal infection and risk for autism in offspring: a meta-analysis. Autism Res. 14, 1296–1316. doi: 10.1002/aur.2499, PMID: 33720503

[ref227] Uekermann J. Kraemer M. Abdel-Hamid M. Schimmelmann B. G. Hebebrand J. Daum I.. (2010). Social cognition in attention-deficit hyperactivity disorder (ADHD). Neurosci. Biobehav. Rev. 34, 734–743. doi: 10.1016/j.neubiorev.2009.10.009, PMID: 19857516

[ref228] Van den Bergh B. R. Mulder E. J. Mennes M. Glover V. (2005). Antenatal maternal anxiety and stress and the neurobehavioural development of the fetus and child: links and possible mechanisms. A review. Neurosci. Biobehav. Rev. 29, 237–258. doi: 10.1016/j.neubiorev.2004.10.007, PMID: 15811496

[ref229] Van Lieshout R. J. Voruganti L. P. (2008). Diabetes mellitus during pregnancy and increased risk of schizophrenia in offspring: a review of the evidence and putative mechanisms. J. Psychiatry Neurosci. 33, 395–404. 18787655 PMC2527714

[ref230] Vigli D. Palombelli G. Fanelli S. Calamandrei G. Canese R. Mosca L.. (2020). Maternal immune activation in mice only partially recapitulates the autism spectrum disorders symptomatology. Neuroscience 445, 109–119. doi: 10.1016/j.neuroscience.2020.05.009, PMID: 32445939

[ref231] Voineagu I. Eapen V. (2013). Converging pathways in autism spectrum disorders: interplay between synaptic dysfunction and immune responses. Front. Hum. Neurosci. 7:738. doi: 10.3389/fnhum.2013.00738, PMID: 24223544 PMC3819618

[ref232] Voineagu I. Wang X. Johnston P. Lowe J. K. Tian Y. Horvath S.. (2011). Transcriptomic analysis of autistic brain reveals convergent molecular pathology. Nature 474, 380–384. doi: 10.1038/nature10110, PMID: 21614001 PMC3607626

[ref233] Vorstman J. A. Parr J. R. Moreno-De-Luca D. Anney R. J. Nurnberger J. I. Jr. Hallmayer J. F. (2017). Autism genetics: opportunities and challenges for clinical translation. Nat. Rev. Genet. 18, 362–376. doi: 10.1038/nrg.2017.4, PMID: 28260791

[ref234] Vuillermot S. Luan W. Meyer U. Eyles D. (2017). Vitamin D treatment during pregnancy prevents autism-related phenotypes in a mouse model of maternal immune activation. Mol. Autism. 8, 1–13. doi: 10.1186/s13229-017-0125-0 28316773 PMC5351212

[ref235] Wallace K. L. Lopez J. Shaffery J. P. Wells A. Paul I. A. Bennett W. A. (2010). Interleukin-10/ceftriaxone prevents E. coli-induced delays in sensorimotor task learning and spatial memory in neonatal and adult Sprague–Dawley rats. Brain Res. Bull. 81, 141–148. doi: 10.1016/j.brainresbull.2009.10.016, PMID: 19883741 PMC2908377

[ref236] Wang Y. Zhang Y. Liu L. L. Cui J. F. Wang J.. (2017). A meta-analysis of working memory impairments in autism spectrum disorders. Neuropsychol. Rev. 27, 46–61. doi: 10.1007/s11065-016-9336-y, PMID: 28102493

[ref237] Weber-Stadlbauer U. Meyer U. (2019). Challenges and opportunities of a-priori and a-posteriori variability in maternal immune activation models. Curr. Opin. Behav. Sci. 28, 119–128. doi: 10.1016/j.cobeha.2019.02.006

[ref238] Webster J. P. Kaushik M. Bristow G. C. McConkey G. A. (2013). Toxoplasma gondii infection, from predation to schizophrenia: can animal behaviour help us understand human behaviour? J. Exp. Biol. 216, 99–112. doi: 10.1242/jeb.074716, PMID: 23225872 PMC3515034

[ref239] Weinstock M. (2005). The potential influence of maternal stress hormones on development and mental health of the offspring. Brain Behav. Immun. 19, 296–308. doi: 10.1016/j.bbi.2004.09.006, PMID: 15944068

[ref240] Weinstock M. (2008). The long-term behavioural consequences of prenatal stress. Neurosci. Biobehav. Rev. 32, 1073–1086. doi: 10.1016/j.neubiorev.2008.03.002, PMID: 18423592

[ref241] Wendeln A. C. Degenhardt K. Kaurani L. Gertig M. Ulas T. Jain G.. (2018). Innate immune memory in the brain shapes neurological disease hallmarks. Nature 556, 332–338. doi: 10.1038/s41586-018-0023-4, PMID: 29643512 PMC6038912

[ref242] Werenberg Dreier J. Nybo Andersen A. M. Hvolby A. Garne E. Kragh Andersen P. Berg-Beckhoff G. (2016). Fever and infections in pregnancy and risk of attention deficit/hyperactivity disorder in the offspring. J. Child Psychol. Psychiatry Allied Discip. 57, 540–548. doi: 10.1111/jcpp.12480, PMID: 26530451

[ref243] Williams P. G. Sears L. L. Allard A. (2004). Sleep problems in children with autism. J. Sleep Res. 13, 265–268. doi: 10.1111/j.1365-2869.2004.00405.x, PMID: 15339262

[ref244] Woods R. M. Lorusso J. M. Potter H. G. Neill J. C. Glazier J. D. Hager R. (2021). Maternal immune activation in rodent models: a systematic review of neurodevelopmental changes in gene expression and epigenetic modulation in the offspring brain. Neurosci. Biobehav. Rev. 129, 389–421. doi: 10.1016/j.neubiorev.2021.07.015, PMID: 34280428

[ref245] Xing D. Oparil S. Yu H. Gong K. Feng W. Black J.. (2012). Estrogen modulates NFκB signaling by enhancing IκBα levels and blocking p65 binding at the promoters of inflammatory genes via estrogen receptor-β. PLoS One 7:e36890. doi: 10.1371/journal.pone.0036890, PMID: 22723832 PMC3378567

[ref246] Xue P. Zheng M. Gong P. Lin C. Zhou J. Li Y.. (2015). Single Administration of Ultra-low-Dose Lipopolysaccharide in rat early Pregnancy induces TLR4 activation in the placenta contributing to preeclampsia. PLoS One 10:e0124001. doi: 10.1371/journal.pone.0124001, PMID: 25853857 PMC4390151

[ref247] Yang Y. Zhao S. Zhang M. Xiang M. Zhao J. Chen S.. (2022). Prevalence of neurodevelopmental disorders among US children and adolescents in 2019 and 2020. Front. Psychol. 13:997648. doi: 10.3389/fpsyg.2022.997648, PMID: 36507037 PMC9730394

[ref248] Yee N. Ribic A. de Roo C. C. Fuchs E. (2011). Differential effects of maternal immune activation and juvenile stress on anxiety-like behaviour and physiology in adult rats: no evidence for the “double-hit hypothesis”. Behav. Brain Res. 224, 180–188. doi: 10.1016/j.bbr.2011.05.040, PMID: 21679729

[ref249] Zablotsky B. Black L. I. Maenner M. J. Schieve L. A. Danielson M. L. Bitsko R. H.. (2019). Prevalence and trends of developmental disabilities among children in the United States: 2009–2017. Pediatrics 144:e20190811. doi: 10.1542/peds.2019-0811, PMID: 31558576 PMC7076808

[ref250] Zaretsky M. V. Alexander J. M. Byrd W. Bawdon R. E. (2004). Transfer of inflammatory cytokines across the placenta. Obstet. Gynecol. 103, 546–550. doi: 10.1097/01.AOG.0000114980.40445.83, PMID: 14990420

[ref251] Zawadzka A. Cieślik M. Adamczyk A. (2021). The role of maternal immune activation in the pathogenesis of autism: a review of the evidence, proposed mechanisms and implications for treatment. Int. J. Mol. Sci. 22:11516. doi: 10.3390/ijms222111516, PMID: 34768946 PMC8584025

[ref252] Zengeler K. E. Lukens J. R. (2021). Innate immunity at the crossroads of healthy brain maturation and neurodevelopmental disorders. Nat. Rev. Immunol. 21, 454–468. doi: 10.1038/s41577-020-00487-7, PMID: 33479477 PMC9213174

[ref253] Zerbo O. Iosif A. M. Walker C. Ozonoff S. Hansen R. L. Hertz-Picciotto I. (2013). Is maternal influenza or fever during pregnancy associated with autism or developmental delays? Results from the CHARGE (childhood autism risks from genetics and environment) study. J. Autism Dev. Disord. 43, 25–33. doi: 10.1007/s10803-012-1540-x, PMID: 22562209 PMC3484245

[ref254] Zerbo O. Qian Y. Yoshida C. Fireman B. H. Klein N. P. Croen L. A. (2017). Association between influenza infection and vaccination during pregnancy and risk of autism spectrum disorder. JAMA Pediatr. 171:e163609. doi: 10.1001/jamapediatrics.2016.3609, PMID: 27893896

[ref255] Zerbo O. Qian Y. Yoshida C. Grether J. K. Van de Water J. Croen L. A. (2015). Maternal infection during pregnancy and autism spectrum disorders. J. Autism Dev. Disord. 45, 4015–4025. doi: 10.1007/s10803-013-2016-3, PMID: 24366406 PMC4108569

[ref256] Zhan Y. Paolicelli R. C. Sforazzini F. Weinhard L. Bolasco G. Pagani F.. (2014). Deficient neuron-microglia signaling results in impaired functional brain connectivity and social behavior. Nat. Neurosci. 17, 400–406. doi: 10.1038/nn.3641, PMID: 24487234

[ref257] Zhou Y. Y. Zhang W. W. Chen F. Hu S. S. Jiang H. Y. (2021). Maternal infection exposure and the risk of psychosis in the offspring: a systematic review and meta-analysis. J. Psychiatr. Res. 135, 28–36. doi: 10.1016/j.jpsychires.2020.12.065, PMID: 33445058

[ref258] Zuloaga D. G. Zuloaga K. L. Hinds L. R. Carbone D. L. Handa R. J. (2014). Estrogen receptor β expression in the mouse forebrain: age and sex differences. J. Comp. Neurol. 522, 358–371. doi: 10.1002/cne.23400, PMID: 23818057 PMC4815281

